# Transcriptome-Wide Association Study of Blood Cell Traits in African Ancestry and Hispanic/Latino Populations

**DOI:** 10.3390/genes12071049

**Published:** 2021-07-08

**Authors:** Jia Wen, Munan Xie, Bryce Rowland, Jonathan D. Rosen, Quan Sun, Jiawen Chen, Amanda L. Tapia, Huijun Qian, Madeline H. Kowalski, Yue Shan, Kristin L. Young, Marielisa Graff, Maria Argos, Christy L. Avery, Stephanie A. Bien, Steve Buyske, Jie Yin, Hélène Choquet, Myriam Fornage, Chani J. Hodonsky, Eric Jorgenson, Charles Kooperberg, Ruth J. F. Loos, Yongmei Liu, Jee-Young Moon, Kari E. North, Stephen S. Rich, Jerome I. Rotter, Jennifer A. Smith, Wei Zhao, Lulu Shang, Tao Wang, Xiang Zhou, Alexander P. Reiner, Laura M. Raffield, Yun Li

**Affiliations:** 1 Department of Genetics, University of North Carolina at Chapel Hill, Chapel Hill, NC 27514, USA; jia_wen@med.unc.edu (J.W.); meximus@126.com (M.X.); laura_raffield@unc.edu (L.M.R.); 2 Department of Biostatistics, University of North Carolina at Chapel Hill, Chapel Hill, NC 27599, USA; bryce.rowland@unc.edu (B.R.); jdrosen@live.unc.edu (J.D.R.); quansun@live.unc.edu (Q.S.); jiawenn@email.unc.edu (J.C.); altapia@live.unc.edu (A.L.T.); madeline.kowalski@nyulangone.org (M.H.K.); yshan@live.unc.edu (Y.S.); 3 Department of Statistics and Operations Research, University of North Carolina at Chapel Hill, Chapel Hill, NC 27599, USA; hjqian@alumni.unc.edu; 4 Department of Epidemiology, University of North Carolina at Chapel Hill, Chapel Hill, NC 27516, USA; kristin.young@unc.edu (K.L.Y.); migraff@email.unc.edu (M.G.); christy_avery@unc.edu (C.L.A.); kari_north@unc.edu (K.E.N.); 5 Division of Epidemiology and Biostatistics, University of Illinois at Chicago, Chicago, IL 60612, USA; argos@uic.edu; 6 Division of Public Health Sciences, Fred Hutchinson Cancer Research Center, Seattle, WA 98109, USA; sbien@fredhutch.org (S.A.B.); clk@fredhutch.org (C.K.); 7 Department of Statistics, Rutgers University, Piscataway, NJ 08854, USA; buyske@stat.rutgers.edu; 8 Division of Research, Kaiser Permanente Northern California, Oakland, CA 94612, USA; Jie.Yin@kp.org (J.Y.); Helene.Choquet@kp.org (H.C.); 9 Institute of Molecular Medicine, McGovern Medical School, The University of Texas Health Science Center, Houston, TX 77030, USA; Myriam.Fornage@uth.tmc.edu; 10 Center for Public Health Genomics, University of Virginia, Charlottesville, VA 22908, USA; ch2um@virginia.edu (C.J.H.); ssr4n@virginia.edu (S.S.R.); 11 Regeneron Genetics Center, Tarrytown, NY 10591, USA; eric.jorgenson@regeneron.com; 12 The Charles Bronfman Institute for Personalized Medicine, Icahn School of Medicine at Mount Sinai, New York, NY 10029, USA; ruth.loos@mssm.edu; 13 Molecular Physiology Institute, Duke University, Durham, NC 27701, USA; yongmei.liu@duke.edu; 14 Department of Epidemiology & Population Health, Albert Einstein College of Medicine, Bronx, NY 10461, USA; jee-young.moon@einsteinmed.org (J.-Y.M.); tao.wang@einsteinmed.org (T.W.); 15 The Institute for Translational Genomics and Population Sciences, Department of Pediatrics, The Lundquist Institute for Biomedical Innovation at Harbor-UCLA Medical Center, Torrance, CA 90502, USA; jrotter@lundquist.org; 16 Department of Epidemiology, School of Public Health, University of Michigan, Ann Arbor, MI 48109, USA; smjenn@umich.edu (J.A.S.); zhaowei@umich.edu (W.Z.); 17 Department of Biostatistics, School of Public Health, University of Michigan, Ann Arbor, MI 48109, USA; shanglu@umich.edu (L.S.); xzhousph@umich.edu (X.Z.); 18 Department of Epidemiology, University of Washington, Seattle, WA 98195, USA; apreiner@uw.edu

**Keywords:** TWAS (transcriptome-wide association study), non-European populations, ancestry, expression analysis

## Abstract

Background: Thousands of genetic variants have been associated with hematological traits, though target genes remain unknown at most loci. Moreover, limited analyses have been conducted in African ancestry and Hispanic/Latino populations; hematological trait associated variants more common in these populations have likely been missed. Methods: To derive gene expression prediction models, we used ancestry-stratified datasets from the Multi-Ethnic Study of Atherosclerosis (MESA, including *n* = 229 African American and *n* = 381 Hispanic/Latino participants, monocytes) and the Depression Genes and Networks study (DGN, *n* = 922 European ancestry participants, whole blood). We then performed a transcriptome-wide association study (TWAS) for platelet count, hemoglobin, hematocrit, and white blood cell count in African (*n* = 27,955) and Hispanic/Latino (*n* = 28,324) ancestry participants. Results: Our results revealed 24 suggestive signals (*p* < 1 × 10^−4^) that were conditionally distinct from known GWAS identified variants and successfully replicated these signals in European ancestry subjects from UK Biobank. We found modestly improved correlation of predicted and measured gene expression in an independent African American cohort (the Genetic Epidemiology Network of Arteriopathy (GENOA) study (*n* = 802), lymphoblastoid cell lines) using the larger DGN reference panel; however, some genes were well predicted using MESA but not DGN. Conclusions: These analyses demonstrate the importance of performing TWAS and other genetic analyses across diverse populations and of balancing sample size and ancestry background matching when selecting a TWAS reference panel.

## 1. Introduction

Hematological measures (red blood cell, white blood cell, and platelet traits) play critical roles in oxygen transport, immunity, infection, thrombosis, and hemostasis and are associated with many chronic diseases, including autoimmunity, asthma, cardiovascular disease, and viral infections. Hematological traits vary between self-reported race/ethnicity groups [1,2,3], in part due to variants which vary in frequency by genetic ancestry group [4,5]. For example, selective pressure from malaria has led to an increased prevalence of sickle cell anemia, G6PD deficiency, and alpha thalassemia in exposed populations [6,7], with impacts on hematological indices [8] in individuals with genetic ancestry from malaria endemic regions. Unfortunately, most existing genome-wide association studies (GWAS) have focused on European ancestry (EA) populations [9,10,11]. It is essential to also explore hematological trait genetics in underrepresented admixed African (AA) and Hispanic/Latino (HL) populations. Realizing that existing genetic studies are failing on diversity [12], multiple consortia have been formed to better represent non-European or multi-ethnic populations. These efforts include the Human Heredity and Health in Africa (H3Africa) consortium [13], and the NIH All of Us program. We anticipate such initiatives improve genomics studies in diverse populations.

Along with the lack of diversity in included populations, GWAS of hematological traits, similar to other complex phenotypes, also identify regions of associated variants whose biological function and target genes are often not clear. One approach to linking variants to genes (and thus to biological function) that has demonstrated success in various complex traits is transcriptome-wide association studies (TWAS) [14,15]. TWAS methods leverage reference expression quantitative trait loci (eQTL) datasets to select genetic variants which in aggregate associate with to predict tissue-specific gene expression. Weights based on variant associations with a transcript are applied to cohorts with genotype (but not expression) data available [15,16]. Transcripts whose levels can be confidently imputed from genetic variants are then assessed for phenotype associations [17]. However, like GWAS, TWAS analyses have often included only EA populations [18,19], though some recent efforts have included more diverse populations [20,21,22,23,24]. Furthermore, most reference eQTL datasets used for TWAS include predominantly EA American individuals [25,26], limiting the predictive power of this novel method in other populations with different allele frequencies.

We examine here the utility of TWAS methods to understand previously identified GWAS loci for blood cell traits, as well as to identify new candidate loci and genes. We focus on underrepresented AA and HL populations and evaluate whether use of relevant ancestry-specific eQTL reference panels from the Multi-Ethnic Study of Atherosclerosis (MESA) improves power for blood cell trait genetic discovery (above primarily European TWAS reference panels).

## 2. Materials and Methods

**Figure 1:**
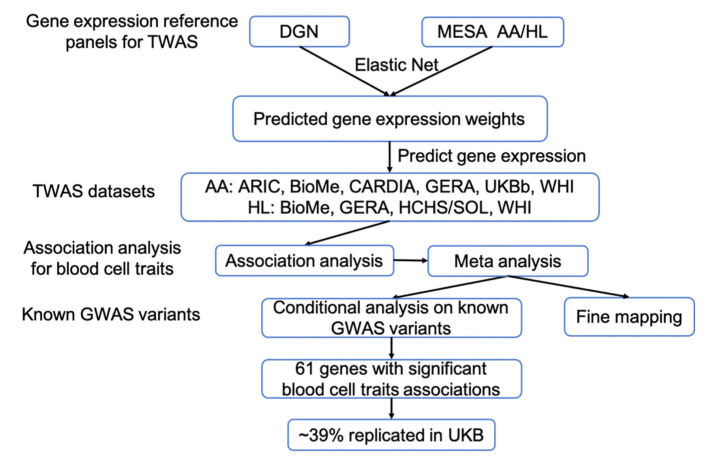
Study design for blood cell trait focused TWAS analyses in African ancestry (AA) and Hispanic/Latino (HL) populations. TWAS: Transcriptome-wide association study; DGN: Depression Genes and Networks, *n* = 922; MESA: Multi-Ethnic Study of Atherosclerosis, *n* = 229 for AA and *n* = 381 for HL; ARIC: Atherosclerosis Risk in Communities, *n* = 2874; BioMe: BioMe^TM^ Biobank, *n* = 3550 for AA and *n* = 4730 for HL; CARDIA: Coronary Artery Risk Development in Young Adults, *n* = 953; GERA: Genetic Epidemiology Research on Adult Health and Aging, *n* = 3699 for AA and *n* = 7348 for HL; UKB: UK Biobank, *n* = 8262; WHI: Women’s Health Initiative, *n* = 8617 for AA and *n* = 4359 for HL; HCHS/SOL: Hispanic Community Health Study/Study of Latinos, *n* = 11,887.

We used three different eQTL datasets, namely Depression Genes and Networks (DGN), MESA AA, and MESA HL, to train gene expression models and then performed association analysis for AA and HL populations and then replicated the results in larger UKB (UK Biobank) EA populations. The study overview is showed in Figure 1 with a more verbose version in Supplementary Figure S1.

### 2.1. Training of TWAS Prediction Models

**Genotype Data.** Using genotype data from 6 African ancestry cohorts (ARIC, BioMe, CARDIA, GERA, UK Biobank, and WHI) and 4 Hispanic/Latino cohorts (BioMe, GERA, WHI, HCHS/SOL) (Figure 1, Supplementary Table S1, Supplementary Content), we performed genotype imputation to the TOPMed freeze 5b reference panel (September 2017 release, methods described at https://www.nhlbiwgs.org/topmed-whole-genome-sequencing-project-freeze-5b-phases-1-and-2, accessed December 2019 and January 2020) on the Michigan Imputation Server [27]. After genotype imputation, we selected variants for TWAS model training if they were well-imputed in all GWAS cohorts and in reference expression quantitative trait loci (eQTL) datasets (MAF > 0.05, *R*^2^ > 0.3). The only exception was for the MESA eQTL reference panel, where direct sequencing data from TOPMed freeze 8 was available for those with monocyte expression microarray data. In brief, the sequencing for MESA was performed with mean genome coverage ≥ 30x and completed at seven sequencing centers; sequencing data files were transferred from sequencing centers to the TOPMed Informatics Research Center (IRC), where reads were aligned to human genome build GRCh38 using a common pipeline across all centers, followed by joint genotype calling. Additional sample-level quality control (such as detection of sex mismatches, pedigree discrepancies, sample swaps, etc.) was undertaken by the Sequencing Centers, the IRC, and the TOPMed Data Coordinating Center (DCC). Detailed methods for freeze 8 are available at https://www.nhlbiwgs.org/topmed-whole-genome-sequencing-methods-freeze-8 (accessed January 2020). For MESA, we included all variants with MAF > 0.05 for TWAS model training. After quality control exclusions, there were 4,694,075 variants in DGN, 4,948,702 variants in MESA AA ancestry, and 4,265,631 in MESA HL ancestry for TWAS modelling. We ran RFMix [28] to infer the global ancestry for all samples across all cohorts. Results are summarized in Supplementary Figure S2 to show the distribution and spectrum of ancestry proportions across cohorts (Supplementary Content).

**Gene Expression Data.** DGN RNA-seq (whole blood) data were obtained from [26]. RNA-seq was already normalized using the Hidden Covariates with Prior (HCP) method [29], correcting for technical and biological factors [19]. A total of 15,231 genes were used for training models in the DGN reference panel. The MESA monocyte gene expression data were obtained from the Illumina HumanHT-12 V4.0 expression beadchip (GSE56045) [30,31,32]. Illumina IDs were converted to Ensembl IDs. For Illumina IDs with multiple Ensembl IDs, each Illumina ID was labeled and treated as an individual gene, leading to multiple TWAS weights being used for 11,056 probe sets (including 8623 genes). More genes were available for TWAS prediction in DGN versus MESA, likely due to a use of RNA-seq instead of expression microarray, larger sample size, and inclusion of additional cell types in whole blood versus monocytes alone (for example, lymphocyte-specific transcripts). To adjust for confounders and then normalize the MESA expression data, we fit a multivariate linear model for gene expression values in MESA controlling for the following covariates: sex, age, age squared, 10 genotype principal components calculated by PC-AiR method from TOPMed freeze 8 WGS data, and 10 PEER factors from microarray gene expression profiling calculated using the “peer” package in R (v 1.0) [33]. Adjusted gene expression values were computed by inverse-quantile normalizing the residuals of the above multivariate linear model.

**Model Training** We used the DGN EA, MESA AA, and MESA HL eQTL datasets to train gene expression prediction models using elastic net. For each gene, variants located within +/− 1Mb of genes start and end were used in model training. Elastic-net methods have been previously shown to be effective even when there is collinearity due to variants in high linkage disequilibrium (LD), so no LD pruning of variants was conducted prior to elastic net model fitting, except for removal of perfectly correlated variants. Hyperparameters were trained via 10-fold cross validation, similar to the PrediXcan approach [15]. Genes with model *R*^2^ ≥ 0.05 for elastic net prediction models were carried forward for TWAS analyses. After model training, we separately applied DGN, MESA AA, and MESA HL training models to predict gene expression values in each GWAS cohort.

These model training methods are essentially the same as the well-established PrediXcan method (elastic net), for which precomputed weights are already available for DGN and MESA at http://predictdb.org/ (accessed January 2020), but we retrained models using TOPMed freeze 5b imputed data and TOPMed freeze 8 WGS data (in MESA) due to substantial gains in imputation quality in AA and Hispanic/Latino populations versus commonly used reference panels like 1000 Genomes [34].

### 2.2. Assessment of Trained Expression Prediction Models in GENOA

To assess the performance of the gene expression prediction models trained from the eQTL reference datasets in an independent AA cohort, we applied the gene expression prediction models to *n* = 802 participants from the Genetic Epidemiology Network of Arteriopathy (GENOA) study [35]. GENOA gene expression was measured in lymphoblastoid cell lines (LCLs) using the Affymetrix Human Transcriptome Array 2.0. We used correlation between real and predicted gene expression transcript values (true *R*^2^) to compare the performance of the MESA AA and DGN TWAS reference datasets.

Based on initial findings that, overall, DGN gene expression prediction was better than the prediction results from the smaller, ancestry-matched MESA cohort, we randomly subsampled DGN to the same size as MESA to evaluate the impact of sample size on the training models. In order to account for variability in prediction accuracy, we randomly performed five subsamplings of the DGN cohort to the MESA cohort sample size. We then retrained TWAS models with elastic net within this smaller subsampled DGN cohort, using the same TWAS training methods described above, and then compared the average of true *R*^2^ from the multiple subsampled-DGN training models with true *R*^2^ from MESA AA (to evaluate if ancestry matching would improve TWAS performance in the same sample size).

### 2.3. Phenotype

We analyzed four blood cell traits from each major hematological domain (hemoglobin—HGB, hematocrit—HCT, white blood cell count—WBC, platelet counts—PLT). These traits had the largest sample size across included cohorts. Prior to association analyses, we excluded extreme outlier values, notably WBC values > 200 × 10^9^/L, HCT > 60%, and HGB > 20 g/dL. For longitudinal cohort studies, all values are from the same exam cycle, with the exam which maximized available sample size chosen (usually baseline). WBC was log transformed due to the skewed distribution. All four traits were adjusted for age, age squared, sex, and top 10 principal components/study specific covariates. The residuals were then inverse normalized.

### 2.4. TWAS Association and Conditional Analysis

We assessed association between predicted gene expression and the inverse normalized HCT, HGB, WBC, and PLT residuals using the cGWAS.emmax function of R package cpgen v0.1, adjusting for an EPACTS kinship matrix. Since linear mixed model allows relatedness among samples by incorporating the kinship matrix, we didn’t prune study samples based on genetic relatedness. Additionally, WBC analyses included adjustment for the Duffy (rs2814778) variant, which strongly impacts neutrophil counts and overall WBC, with the null allele much more common in African ancestry populations [4]. Following association testing within each cohort, we performed meta-analysis within ancestry groups using METAL [36] We further investigated genes with a nominal meta-analysis *p*-value < 1 × 10^−4^ through a conditional association test on all known variants within +/− 1 Mb flanking regions. Strict Bonferroni significance thresholds (0.05/# tested genes) are listed in Supplementary Table S7. We performed conditional analysis by conditioning on these known signals within +/− 1 Mb of each significant gene in two steps (Supplementary Table S5). We additionally aggregated four sets (i.e., DGN AA, DGN HL, MESA AA, MESA HL) of *p*-values using the Cauchy combination test implemented by ACAT [37]. We would like to note that most meta-analysis approaches including ACAT may be sub-optimal for detecting ancestry specific associations. For example, the *TNFAIP2*-platelets association highlighted was only discovered by MESA HL (because this gene was not available in the other three sets: poorly predicted by DGN and not even present in MESA AA), and therefore, not surprisingly, was not Bonferroni significant after ACAT aggregation. We initially conducted conditional analysis using known variants from the GWAS catalog as of June 2018 (Step 1). If the TWAS signal remained significant after Step 1 conditional analysis, or if the TWAS signal had no GWAS catalog GWAS variants within +/− 1 Mb region, we further conditioned on significant (*p* < 5 × 10^−8^) variants from recent blood cell traits GWAS [11,38] (Step 2). We then keep the larger (i.e., less significant) conditional *p*-value as the final results. Note that we used a lenient threshold of 0.05 to declare conditional significance. In addition, we define a locus from our TWAS results as the +/− 1 Mb region of most significant sentinel gene’s start and end positions. For loci with more than one marginally significant gene identified, we further perform fine-mapping using FINEMAP [39].

### 2.5. Replication

Our replication procedure was split into two tiers of testing to assess the effect of ancestry-matched gene expression prediction models in TWAS replication. First, we attempted replication using gene expression prediction models trained in DGN, but further restricted to variants that are common and well-imputed (MAF > 0.05, *R*^2^ > 0.8) in both DGN and UKB Europeans (DGN-for-UKB). We retrained gene expression prediction model using DGN-for-UKB with the same elastic net procedure described above.

For genes with models trained in DGN using variants available in all cohorts in the AA or HL TWAS meta-analysis dataset, DGN-for-UKB trained models served as the only model for predicted gene expression considered in initial replication analyses. For MESA AA or MESA HL trained models, if either the gene-trait association was not significant at the Bonferroni adjusted threshold (*p* < 8 × 10^−4^) or the prediction quality of the gene expression prediction model was poor (model *R*^2^ < 0.01), we used the MESA trained model to impute gene expression values into UKB Europeans, in a second tier of replication analyses.

Sixty-one gene-trait pairs that demonstrated evidence of conditionally significant association in AA or HL TWAS meta-analyses were included in the UK Biobank replication analysis. We used a Bonferroni-corrected threshold for the number of tests (0.05/61 = 8 × 10^−4^) to identify replicated signals. Phenotypes were adjusted for covariates and inverse normalized, as described above for AA and HL cohorts (additional details in Supplemental Content, Included Cohorts). REGENIE [40] was used to test the association between predicted gene expression and adjusted phenotypes.

## 3. Results

### 3.1. Train Gene Expression Prediction Models from Reference eQTL Datasets

**Figure 2:**
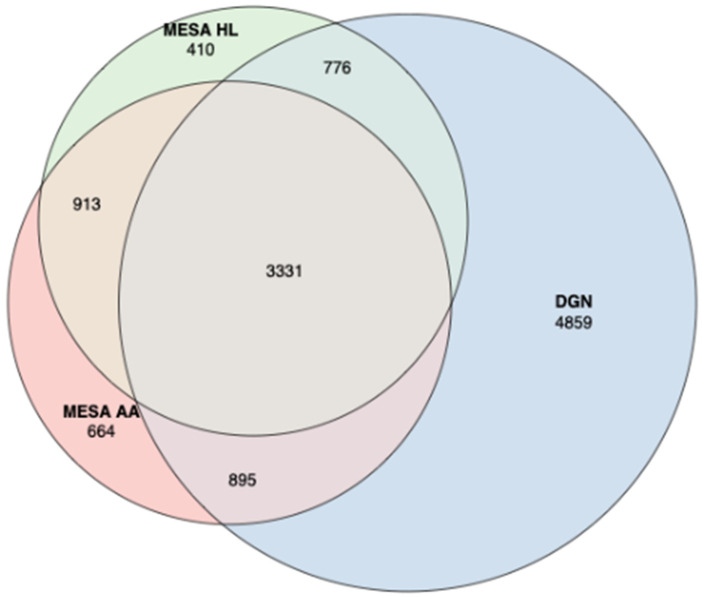
Venn diagram showing the overlap of well-predicted genes by Depression Genes and Networks (DGN) European ancestry and Multi-Ethnic Study of Atherosclerosis (MESA) African ancestry (AA) and Hispanic/Latino (HL) reference panels.

**Figure 3:**
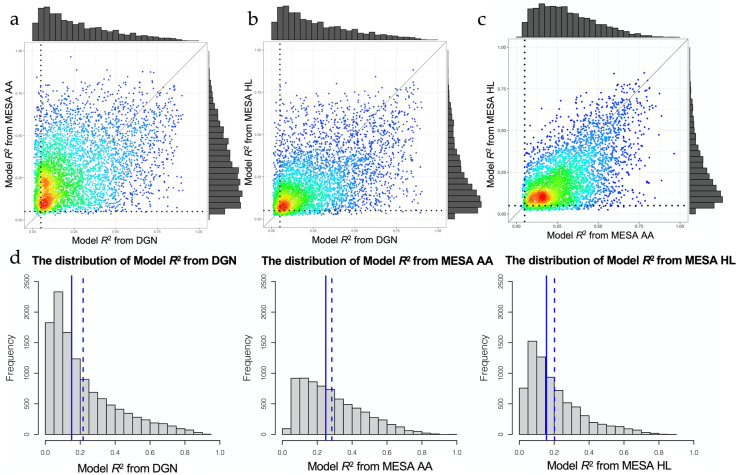
The smooth scatter plots show the model *R*^2^ distribution of common genes available in both the Depression Genes and Networks (DGN) European and Multi-Ethnic Study of Atherosclerosis (MESA) reference eQTL datasets. The dashed line denotes the threshold value (model *R*^2^ = 0.05) for well-predicted genes. (**a**) Comparison of model *R*^2^ of common genes found in both Depression Genes and Networks (DGN) and Multi-Ethnic Study of Atherosclerosis (MESA) African ancestry (AA) reference panels; (**b**) Comparison of model *R*^2^ of common genes between the Depression Genes and Networks (DGN) and Multi-Ethnic Study of Atherosclerosis (MESA) Hispanic/Latino (HL) reference panels; (**c**) Comparison of model *R*^2^ of common genes between the Multi-Ethnic Study of Atherosclerosis (MESA) African ancestry (AA) and Multi-Ethnic Study of Atherosclerosis (MESA) Hispanic/Latino (HL) reference panels; (**d**) Histograms showing the model *R*^2^ distribution of all genes in each reference panel (without model *R*^2^ filtering). The blue solid line denotes the median of model *R*^2^; the blue dashed line denotes the mean of model *R*^2^. All genes, including those which do not meet a model *R*^2^ = 0.05 cut-off, are displayed.

In this study, we leveraged data from two studies as reference eQTL datasets: EA individuals from the Depression Genes and Networks (DGN) cohort (*n* = 922 with whole blood RNA-sequencing data [26]), and AA (*n* = 229) and HL (*n* = 381) individuals from the MESA study [30,31]. We attempted to train gene expression prediction models using elastic net for 11,687 genes, 5958 genes with 7120 probe sets, and 6082 genes with 7316 probe sets in DGN EA, MESA AA, and MESA HL eQTL reference panels, respectively. Following previous studies [15,16], we retained prediction models with model *R*^2^ > 0.05 for 9861 genes, 5883 genes with 7026 probe sets, and 5522 genes with 6559 probe sets, respectively. From the training results, based on model *R*^2^ > 0.05, 3331 genes were well-predicted with all reference panels, but 4859 were well-predicted only with DGN (and 664 and 410 only in MESA AA and HL, respectively, Figure 2). If we restrict to the 3931 genes present in both the DGN and both MESA reference panels, 3927 genes are well-predicted (model *R*^2^ > 0.05) by at least one of the three reference panels; 7 genes are well predicted only with DGN; 50 only with MESA AA; and 5 only with MESA HL (Supplementary Figure S1). Comparing the distribution of model *R*^2^ across all genes and common genes, the MESA AA reference panel performed slightly better than DGN, while DGN performed slightly better than MESA HL (Figure 3, Supplementary Table S2). The large number of genes (4859) only well-predicted with DGN is likely due to the larger sample size and higher number of expressed genes for this reference panel, as well as the greater number of included cell types in whole blood. Genes not highly expressed in monocytes are unlikely to be well predicted with the MESA reference panel. Differences in model *R*^2^ across reference panels can be quite striking; for example, LTBP3 is well predicted in DGN (model *R*^2^ = 0.47), but not well predicted with MESA HL (model *R*^2^ = 0.03), and less than 2 non-zero weights contribute to the LTBP3 prediction model with MESA AA. The Human Protein Atlas, a database that provides gene expression in different blood cell types, shows that LTBP3 has higher gene expression in lymphocytes such as the memory B-cells and naive B-cells but very low gene expression in myeloid-lineage white blood cell types such as monocytes, eosinophils, and neutrophils [41], which may explain this poor prediction using MESA monocyte expression data as a reference panel. However, for a subset of genes, the MESA ancestry-specific reference panels had better performance, such as TNFAIP2 with model *R*^2^ = 0.10 in MESA HL but only *R*^2^ = 0.02 in DGN. For TNFAIP2, variants in the prediction models for MESA HLs were more common in HL than EA populations (14/16, 87.5%) using 1000 Genome phase 3 data as a minor allele frequency (MAF) reference.

### 3.2. Assessment of Trained Expression Prediction Models in Independent Non-European Datasets

**Figure 4:**
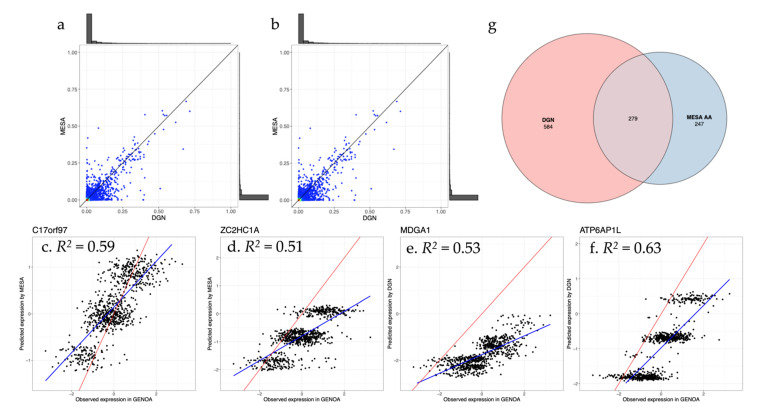
Smooth scatter plots comparing true gene expression from Genetic Epidemiology Network of Arteriopathy (GENOA) lymphoblastoid (LCL) data to predicted gene expression using Depression Genes and Networks (DGN) and Multi-Ethnic Study of Atherosclerosis (MESA) African ancestry (AA) reference eQTL datasets. (**a**) True *R*^2^ distribution using the Depression Genes and Networks (DGN) and Multi-Ethnic Study of Atherosclerosis (MESA) eQTL reference panel for all genes (# genes = 4043); (**b**) true *R*^2^ distribution for genes with model *R*^2^ > 0.05 in each reference eQTL dataset (# genes = 3426); (**c**–**f**). Prediction performance of four examples of well-predicted genes. The scatter plots show the observed expression in GENOA versus predicted expression using prediction models build using MESA AA (**c**,**d**) and DGN (**e**,**f**). The observed expression in GENOA is from array data in LCLs. The red line is the diagonal line with slope = 1 and the blue line is the fitting line between the observed expression and predicted expression; (**g**) Venn diagram of well-predicted genes (true *R*^2^ > 0.05) by DGN and MESA AA reference panels in GENOA.

To assess the performance of gene expression prediction models trained from the eQTL reference datasets among minority participants, we applied TWAS prediction models to AA participants (*n* = 802) from the Genetic Epidemiology Network of Arteriopathy (GENOA) study [35]. We compared the performance of prediction models trained from MESA AA and DGN reference eQTL datasets in terms of the correlation between predicted and true (true *R*^2^) (Figure 4a,b, Supplementary Table S3). When comparing genes found in both DGN and MESA reference panels, we found that DGN performs better than MESA AA in prediction of GENOA expression, based on true *R*^2^ (Figure 4a). Restricting to well-predicted genes in both DGN and MESA AA (model *R*^2^ > 0.05 in both reference panels), DGN outperforms MESA AA as well (Supplementary Table S3, Figure 4b). We then selected four well-predicted genes as examples, two from MESA-AA predicted models and two from DNA predicted models, all illustrated in Figure 4c–f. Figure 4c–f are scatter plots showing the observed expressions in GENOA and predicted expressions using MESA AA (Figure 4c,d) and DGN (Figure 4e,f) as reference, respectively. Among them, 279 genes had true *R*^2^ > 0.05 in GENOA with both reference panels, 584 only with DGN, and 247 only with MESA AA (Figure 4g) and comparison results at different true *R*^2^ threshold values were showed in Supplementary Figure S3. As expected, the proportion of shared well-predicted genes between DGN and MESA AA increases with true *R*^2^ threshold values (Figure 4g, Supplementary Figure S3e). We suspected that DGN might be performing slightly better than the ancestry-matched MESA AA reference due to the larger sample size. We therefore sub-sampled DGN to be the same size as the MESA AA panel and predicted into the external GENOA dataset, to directly compare the impact of sample size on the training model. The true *R*^2^, which decreased with the reduced sample size for the sub-sampled DGN panel, was still slightly better, on average, than models trained from MESA AA (Supplementary Table S3) when restricting to common predicted genes between DGN and MESA AA. The decreased true *R*^2^ of sub-sampled DGN models compared to the original full DGN-sample models does, however, directly demonstrate the effect of sample size on the prediction performance (Supplementary Table S3). In addition, we still found GENOA genes which are well-predicted by MESA but very poorly predicted by DGN, such as *POLR2J*, which is highly expressed in most blood cells (including monocytes and lymphocytes) [41]. For *POLR2J*, true *R*^2^ in GENOA is 0.42 using the MESA TWAS reference panel versus *R*^2^ = 0.007 using DGN as a TWAS reference panel. Comparing the variants included in prediction models from both reference panels, 39 of 57 variants (~68%) used in MESA AA derived prediction model are more common in AA than EA populations, which may lead to better prediction, while only 4 of 27 (~15%) included variants are more common in AA versus EA populations for the DGN prediction model. Allele frequency differences across populations likely leads to the lower true *R*^2^ using DGN as a reference panel.

### 3.3. Association between Predicted Gene Expression and Blood independent Traits

We applied TWAS weights from both DGN and MESA to independent cohorts of AA ancestry (*n* = 27,955) and HL (*n* = 28,324) participants and performed TWAS for four blood cell traits: HGB, HCT, WBC, and PLT. We selected these four traits to both encompass all three major hematological domains (red and white blood cells, and platelets) and retain maximal sample size in each constituent cohort (due to extensive missing data for most other traits). Results were then meta-analyzed for each trait within each ancestry group (AA or HL).

For the 27,955 AA participants from 6 AA cohorts (Supplementary Content and Supplementary Table S1), we conducted TWAS with the four hematological traits using pre-trained models on 9861 and 5883 genes with 7026 probe sets from the DGN and MESA AA eQTL reference dataset, respectively. For the 28,324 HL participants from 4 HL cohorts (cohort details in Supplementary Content and Supplementary Table S1), we performed TWAS with the four blood cell traits for 9861 and 5522 genes with 6559 probe sets trained in DGN and MESA HL eQTL reference datasets, respectively.

**Figure 5:**
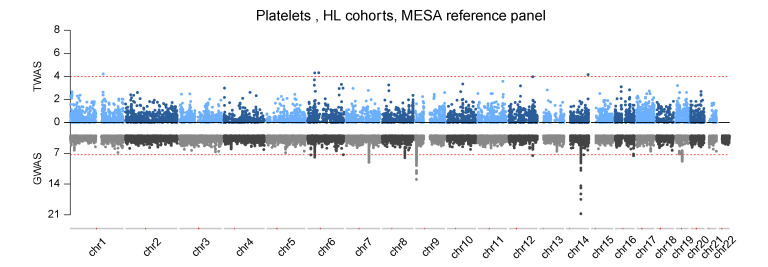
Mirror plot for the TWAS and GWAS results for platelets (PLT) in Multi-Ethnic Study of Atherosclerosis (MESA) Hispanic/Latino (HL). The upper panel shows TWAS marginal results and Table 1. $10^{-4}$ for TWAS and 5 $\times 10^{-8}$ for GWAS.

Meta-analyses revealed 42 and 27 marginally significant gene-trait pairs in AA and HL, respectively, when DGN expression prediction models were used. When prediction models from MESA AA and HL samples were employed, 22 and 18 marginally significant gene-trait pairs were identified for AA and HL, respectively (Table 1, Figure 5, Supplementary Figures S1 and S4). Effect sizes of the 65 associations in AA range from -0.312 to 0.381 with the absolute mean of 0.112 across the four blood cell traits for AA. Effect sizes of the 45 associations in HL range from −0.356 to 0.302 with the absolute mean of 0.130 across the four blood cell traits for HL. The largest effect size we observed is from *ABCA13* for WBC, with a value of 0.381, meaning that for every unit change in the expression of *ABCA13*, WBC increases by 0.381 (Supplementary Table S4). Note that the effect sizes are comparable across genes because we inversed-normalized the gene expression values (detailed in Methods 2.1). In total, we identified 80 unique genes and 90 unique gene-trait pairs across AA and HL. Of 90 unique marginally significant gene-trait pairs, we found 13 gene-trait pairs by both DGN and MESA (AA and HL) prediction models, 51 gene-trait pairs by DGN models only, and 26 gene-trait pairs using MESA AA and/or HL prediction models only. We note that there were 23 unique genes associated with blood cell traits only when using non-EA reference panels, despite the much smaller sample sizes compared to DGN EA reference (detailed in Supplementary Table S4, Supplementary Figure S4).

We therefore calculated cross-validation *R*^2^ for the 80 marginally significant genes (i.e., genes that showed suggestive association (*p* < 1 $\times 10^{-4}$) with at least one blood cell trait). Overall, cross-validation *R*^2^ are smaller than model *R*^2^, but overall, reasonably consistent (Pearson correlation between model *R*^2^ and cross-validation *R*^2^ is 0.91 with *p*-value < 2.2 × 10^−16^) (Supplementary Table S4 and Supplementary Figure S5). Importantly, all genes we highlight were reasonably well-predicted based with cross-validation *R*^2^ > 1%.

### 3.4. TWAS Analysis Conditional on Neighboring GWAS Variants

For the 90 marginally significant gene-trait pairs, 67 genes (involved in 74 unique gene-trait associations, ~82.2%) have known GWAS-identified variant associations for the same hematological trait within +/− 1 Mb. We performed conditional analysis to investigate whether the significant TWAS genes remain significant when conditioned on known GWAS variants, which would suggest that additional genetic variants regulating expression of the TWAS identified gene remain to be discovered (see Methods). The conditional analysis shows that 45 (including 42 genes) out of the total 74 (~60.8%) unique associations are still nominally significant when conditioned on known GWAS variants (*p* < 0.05, Supplementary Table S4). An additional 16 out of 90 unique gene-trait associations have no known GWAS variants within 1 Mb. In the replication analysis using the much larger UKB EA sample, we focused on the 42 conditionally significant genes (45 gene-trait associations) and the 15 genes with no known GWAS variants within +/− 1 Mb (16 gene-trait associations), as these were the genes with evidence of association with hematological traits not captured by existing GWAS, for 61 total gene-trait associations (Supplementary Table S4).

### 3.5. Replication in UK Biobank

To validate the conditionally significant gene-trait associations we identified in the discovery cohorts, we performed replication analysis in 405,782 EA participants from the UKB. Specifically, we attempted to validate both the conditionally significant gene-trait associations and gene-trait associations without nearby known GWAS variants. We note that African ancestry UKB participants were already included in our discovery TWAS meta-analyses and thus were not available for replication purposes. Using independently trained DGN-for-UKB gene expression prediction models, 21 out of 49 gene-trait association pairs were successfully replicated at significance level of α < 8 $\times 10^{-4}$ (α = 0.05/61). For the 16 genes (involved in 16 associations) which did not have a valid DGN-for-UKB gene expression prediction model, we performed replication analysis in UKB-EA using the same models for gene expression prediction used in MESA AA and MESA HL. Of these genes, 3 out of 16 replicated in the secondary analysis at α < 8 $\times 10^{-4}$ (α = 0.05/61). We note that almost all of these replicated genes are conditionally distinct signals at known loci; of the 16 unique gene-trait pairs with no nearby GWAS identified variants, only *APEH*’s association with hematocrit in AA populations compellingly replicates in UK Biobank (*p* = 1.7 × 10^−6^). Two findings of interest, both of which replicated in UKB, are described below.

### 3.6. Example Replicated Genes Still Nominally Significant after Conditioning on Known GWAS Variants

**Figure 6:**
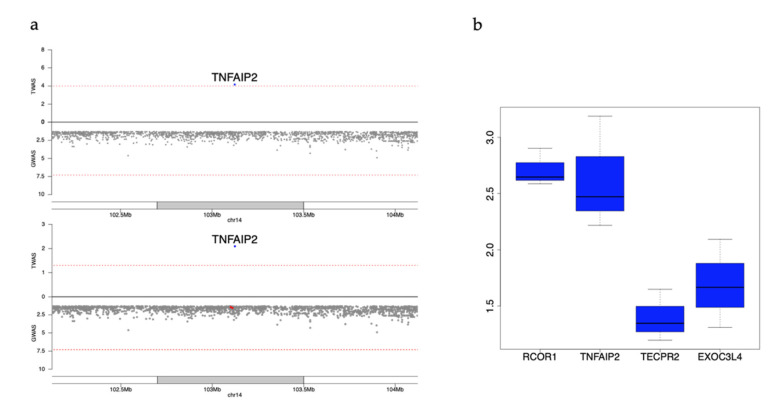
*TNFAIP2* locus. (**a**) Mirror plots showing the conditional analysis for *TNFAIP2*, predicted using the Multi-Ethnic Study of Atherosclerosis (MESA) Hispanic/Latino (HL) reference panel, for platelet association meta-analysis in Hispanic/Latino cohorts. The red dots in the bottom panel denote the nearby GWAS signals conditioned on. *TNFAIP2* is still significant when conditioned on nearby GWAS signals (as listed in Supplementary Table S5); (**b**) Gene expression for *TNFAIP2* and the other three GWAS annotated genes in this locus from platelet-producing megakaryocytes (MK) from BLUEPRINT [43].

***TNFAIP2* (TNF alpha Induced Protein 2)** In the analyses conducted in HL populations using MESA as reference, *TNFAIP2* remains significant for platelet count when conditioned on known GWAS variants (marginal *p* = 6.8 × 10^−5^; conditional *p* = 8.1 × 10^−3^). As noted previously, *TNFAIP2* is not well-predicted using the DGN reference panel; the prediction model of *TNFAIP2* using MESA HL as a reference mostly includes variants more common in HL than EA populations, which may lead to better prediction using this ancestry-matched reference panel. We assessed the variants which contribute to *TNFAIP2* prediction in functional annotation data from megakaryocytes, the cells which produce platelets. Five variants (Variants ID of chr:GRCh38-positions:Ref_allele:Alt_allele: 14:103099825:G:A; 14:103112649:T:C; 14:103118337:A:G; 14:103378293:G:A, and 14:103535180:G:A) used in the MESA HL TWAS prediction model for *TNFAIP2* are annotated as being in open chromatin by megakaryocyte ATAC-seq [42]. Megakaryocyte gene expression from the BLUEPRINT consortium [43] shows that *TNFAIP2* has higher expression in this cell type than other nearby GWAS annotated genes (*EXOC3L4* and *TECPR2*) and has comparable expression with *RCOR1* (Figure 6). Moreover, this gene replicates in the UKB EA cohort using variant weights derived from the MESA HL reference panel (marginal *p* = 2.9 × 10^−30^).

**Figure 7:**
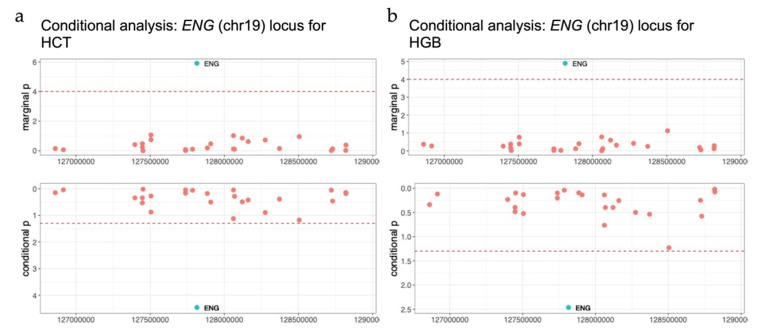
*ENG* locus. (**a**) The marginal and conditional results for *ENG* for hematocrit (HCT) and (**b**) hemoglobin (HGB) predicted using the Multi-Ethnic Study of Atherosclerosis (MESA) reference panel in African ancestry (AA) meta-analysis. The green dot denotes the gene *ENG*, the red dots denote other genes within this locus region.

***ENG* (Endoglin).** *ENG* is a known hemoglobin (HGB)/hematocrit (HCT) associated gene reported in EA populations in previous GWAS [11,38,44]. We identified this gene in our TWAS meta-analyses using weights from the MESA AA panel (marginal *p* = 1.27 × 10^−5^ for HGB, *p* = 1.24 × 10^−6^, which passes the Bonferroni threshold value for HCT), but not the DGN panel (marginal *p* = 5 × 10^−3^ for HGB and *p* = 3 × 10^−4^ for HCT) (Figure 7). When conditioned on known GWAS variants for HGB/HCT, *ENG* is still nominally significant with conditional *p* = 3.44 × 10^−3^ for HGB and *p* = 2.6 × 10^−3^ for HCT. We further investigated the MAF distribution of variants used in the prediction models of the two training panels and found 18/22 (~81.8%) variants used in the MESA-derived prediction model are more common in AA versus EA individuals in 1000 G, with similar enrichment not observed for the DGN EA reference panel weights (27/53 variants (~50.9%) more common in AA populations).

### 3.7. FINEMAP Analysis for Significant Gene-Trait Associations

In total, we identified 66 loci for 4 blood cell traits across AA and HL using both reference panels (Supplementary Table S4), of which 58 loci only include one gene. There are in total 27 gene-trait pairs in the remaining 8 loci which include at least two signals. For prioritizing the candidate causal gene at these loci, we performed fine-mapping using FINEMAP [39], however the findings were difficult to interpret, in particular for the following two examples.

**Figure 8:**
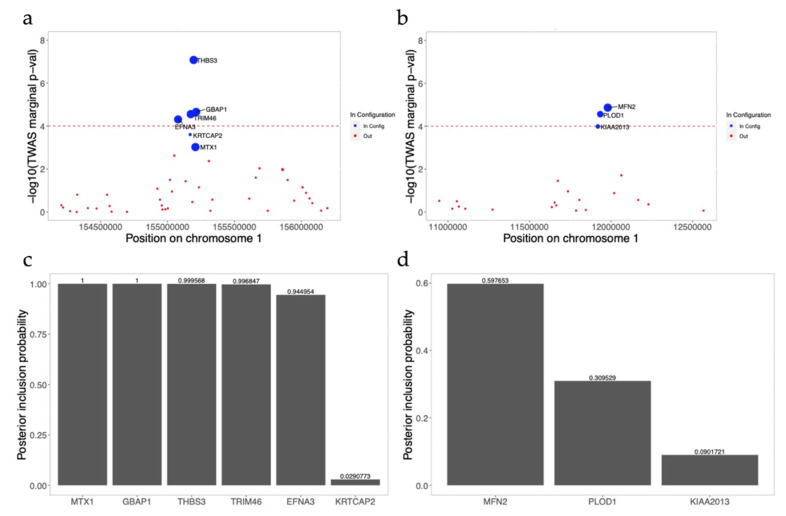
Fine-mapping of the *THBS3* and *MFN2* locus. Blue dots denote genes in the causal gene set configuration; red dots denote the genes outside of the causal gene set configuration. Dot size is proportional to the marginal posterior inclusion probability of each gene in the 95% credible set within the locus. The red dashed line denotes the TWAS significance threshold value. (**a**,**c**) *THBS3* locus for hematocrit using Depression Genes and Networks (DGN) reference panel in African ancestry (AA) and the posterior inclusion probability of each gene in the 95% credible set within this locus; (**b**,**d**) *MFN2* locus for platelet count using Depression Genes and Networks (DGN) reference panel in African ancestry (AA) and the posterior inclusion probability of each gene in the 95% credible set within this locus.

***THBS3* locus.** The *THBS3* locus includes four genes: *THBS3*, *EFNA2*, *TRIM46,* and *GBAP1*. *THBS3* was the most marginally significant gene, passing the Bonferroni threshold value (marginal *p-*value = 9.8 × 10^−8^) for HCT from TWAS analysis using the DGN reference panel in HL. The correlation of predicted gene expression was very low for these 4 genes (correlation *R*^2^ ranged from 7 × 10^−4^ to 0.03). However, the fine-mapping results included 6 genes: 4 significant genes (*EFNA2*, *TRIM46*, *THBS3,* and *GBAP1*) plus 2 additional non-significant genes (*MTX1* and *KRTCAP2*), in the 95% credible set (Figure 8a,c). *MTX1* is reported as the mapped gene for the previously reported GWAS lead variant for HCT at this locus (i.e., rs760077) [9,11,38]. However, *MTX1* was not significant in our TWAS results, despite adequate model *R*^2^ for gene expression prediction (marginal *p* = 0.001; model *R*^2^ = 0.10). Within this locus the predicted gene expression between the lead gene *THBS3* and GWAS-reported gene *MTX1* was not correlated (*R*^2^ = 7.2 × 10^−5^), but the gene prediction correlations are stronger between *MTX1* and *GBAP1* (*R*^2^ = 0.31), *MTX1* and *EFNA3* (*R*^2^ = 0.40), and *MTX1* and *TRIM46* (*R*^2^ = 0.78). We further note that FINEMAP assigned *MTX1* the highest marginal posterior inclusion probability (PIP = 1) [9,44], followed by *GBAP1*. Given the high correlations between *GBAP1*, *EFNA3*, *TRIM46,* and *MTX1*, none of which are correlated with lead TWAS gene (*THBS3*), it is difficult to accurately narrow down a reasonably sized candidate causal gene set at this locus. The low PIP for the most significant *THBS3* gene is also difficult to interpret. In summary, fine-mapping was not informative in pinpointing the candidate causal gene for this locus.

***MFN2* locus.** The *MFN2* locus includes two significant genes: *PLOD1* and *MFN2*. The lead gene *MFN2* is the most significant gene for platelet count in our AA-focused meta-analysis (*p* = 1.36 × 10^−5^, DGN reference panel). *MFN2* is a hemostasis-related gene involved in megakaryocyte development and platelet production [45]. Both of the genes were reported in prior GWAS results based on proximity (the TSS of *MFN2* is ~5 kb from the 3′UTR of *PLOD1* on the same strand) [9,38]. Fine-mapping results reported three genes (*PLOD1*, *KIAA2013,* and *MFN2*) in the 95% credible set at this locus. The predicted expression of the lead gene, *MFN2*, is not highly correlated with *KIAA2013* (*R*^2^ = 0.005) or *PLOD1* (*R*^2^ = 0.003); however, fine-mapping shows that all these three genes are in the 95% credible causal gene set (Figure 8b,d). Additionally, around 50–55% of variants are shared in the three gene prediction models, even though the predicted expression correlations are low, which may contribute to the unclear 95% credible set results.

We further checked additional 6 loci from our TWAS results and found that the 95% credible causal gene sets were very similar: fine-mapping included all marginal significant genes within each locus and did not help to distinguish the candidate causal genes (Supplementary Table S6).

## 4. Discussion

In recent years, we have increasingly realized that our genomic studies are failing on diversity. We observe examples where certain population-specific variants significantly associated with complex traits: examples include the African specific Duffy null variant rs2814778 alone accounts for 15–20% of variability in white blood cell counts among AAs [4,46], and the trypanolytic *APOL1* G1/G2 alleles conferring an estimated ~20% lifetime risk of developing chronic kidney disease [47,48]. In addition, certain drugs may be less effective or even unsafe in some racial/ethnic groups due to genetic differences [12]. For GWAS, studies embracing ethnic diversity have been recognized as more powerful for gene mapping due to differences in allele frequency, effect size, haplotype background of causal variant(s), and LD across ethnicities [49,50], as well as factors that are broadly defined as social determinants of race including but not limited to differential pathogen invasion, environmental exposures, perceived discrimination and associated psychosocial stress [51,52,53]. We therefore consider it a pressing task to increase diversity in our genetic studies.

Here, we conducted large-scale TWAS meta-analyses for 4 blood cell traits in AA and HL populations, using a larger whole-blood eQTL reference panel (DGN) in European ancestry populations and smaller but ancestry-matched reference panels from MESA monocytes. To our knowledge, this is the largest genetic discovery effort yet conducted in these populations for blood cell indices, exceeding available sample sizes from recent trans-ethnic blood cell traits GWAS ([38], *n* = 15,171 African ancestry, *n* = 9368 Hispanic/Latino), recent analyses of red blood cells in the Population Architecture using Genomics and Epidemiology [PAGE] studies ([54], *n* = 16,258 African American, *n* = 20,784 Hispanic/Latino), and our own recent TOPMed imputed GWAS analyses ([55], *n* = 21,513 African ancestry and 21,689 Hispanic/Latino participants). These previous efforts all included some but not all of the cohorts included here. In our comparisons of expression reference panels, using one of the largest expression datasets in African Americans (GENOA LCL data) as a testing dataset for comparing imputed to true gene expression (using the DGN, sub-sampled DGN and MESA TWAS training reference panels), we observed that the larger sample size for DGN (as well as the potentially the use of whole blood instead of monocyte specific data, or RNA-seq versus expression microarray) led to slight improvements in model performance over MESA AAs. The generalizability of these comparisons in GENOA is limited by tissue heterogeneity between the DGN and MESA reference panels, however. However, we see additional significant gene-trait associations using the ancestry-matched monocyte eQTL reference panels not captured using DGN, and these TWAS models generally include a high percentage of variants more common in non-European populations. This is true even for red blood cell and platelet traits; in theory, a monocyte-specific eQTL dataset should be a poorer model for these traits than whole blood, as unlike in whole blood no transcripts from platelets or red blood cells would be included (though even for whole blood, the vast majority of transcripts are for white blood cells (monocytes and lymphocytes)). Finding new, replicated loci in much more modest sample sizes than for current trans-ethnic GWAS efforts (which include > 750,000 participants [38]) also highlights the importance of performing TWAS and other genetic analyses in diverse populations.

Previous work has also suggested the value of ancestry-matched TWAS reference panels. Recent work from Geoffroy, et al. [24] found more significant complex trait-associated genes in summary statistics from the diverse PAGE consortium (~50,000 HL, African American, Asian, Native Hawaiian, and Native American individuals) using models trained in African American and HL MESA participants than in European- or all-ancestry TWAS models. Work in the diverse Carolina Breast Cancer Study (CBCS) cohort also suggests that TWAS models perform poorly across race/ethnicity groups, with multiple novel discoveries for breast cancer survival found in AA women using TWAS gene expression models trained in a subset of the cohort with measured expression data [21]. Poor performance comparing real and predicted gene expression in whole blood was also observed for GTEx v7 (>85% European) and DGN TWAS weights in a pediatric African American cohort—the Study of African Americans, Asthma, Genes, and Environment (SAGE)—with substantial declines in prediction accuracy in African ancestry populations from the GEUVADIS LCL expression datasets also observed versus European ancestry datasets [56]. Sample sizes remain limited for transcriptome data in non-European populations, however. For example, the large blood-based eQTLGen meta-analysis included almost exclusively European-ancestry individuals [57]. Additional data generation in non-European populations is important to improve TWAS performance and gene-trait association discovery.

Our work also identified biologically relevant genes for hematological traits at loci not containing previous GWAS signals, as well as genes which may explain known GWAS signals in gene-dense regions or highlight additional sub-genome-wide significant conditionally distinct signals at known loci, such as *TNFAIP2*. Previous studies reported that *TNFAIP2*, identified by TWAS with the MESA HL reference panel for platelet count, is related to acute promyelocytic leukemia [58,59] and may play a role as mediator of inflammation [60] and angiogenesis [61]. It is also known to be highly expressed in mononuclear progenitor cells of the bone marrow [62] and in mature peripheral blood monocytes [63], and plays a crucial role in apoptosis [60,64,65]. One study reported that *TNFAIP2* was involved with the protective mechanism of Amentoflavone in the hematopoietic system of mice against γ-irradiation [62]. While this broad locus for platelet count was known from GWAS, conditional analysis adjusting for these known GWAS identified variants suggests additional variant-trait associations remain to be discovered, and this gene also has not been commonly identified as the likely target gene in prior GWAS studies [11,38] (where annotated genes were often based on proximity alone). This gene-trait association was not identified using the DGN reference panel, likely due to the allele frequency differences for variants in the gene expression prediction model in European-ancestry and HL populations, further demonstrating the importance of ancestry matched reference panels.

Even though fine-mapping could help to prioritize the candidate causal genes at TWAS loci in some cases, our results show that fine-mapping may not always provide assistance in identifying candidate causal genes. There are several challenges for TWAS fine-mapping. One challenge is that shared eQTLs in the prediction models for multiple genes could bias the fine-mapping results, such as those observed at the *MFN2* locus. Having a large number of genes whose predicted expression is highly correlated to the GWAS reported gene could also bias fine-mapping results, as we observed at the *THBS3* locus. Moreover, FINEMAP, which performs fine-mapping using the correlation matrix based on the gene prediction models, may not reflect the true correlation of gene expression within a locus. Future work on pinpointing the potential causal genes from TWAS may include using measured gene expression, instead of predicted gene expression, to calibrate the correlation matrix in fine-mapping analysis [14].

Our analysis has a number of limitations. First, our TWAS significance threshold value was lenient. Using a strict Bonferroni (0.05/# tested genes, Supplementary Table S7) significance threshold, only 24 out of 90 unique gene-trait associations noted in Supplementary Table S4 (~26.7%) met that criterion, most of which are well known genes from previous GWAS studies. To increase our odds of identifying novel and biologically interesting signals in understudied AA and HL populations, we chose 1 × 10^−4^ as a nominal significance threshold, but only considered as true findings genes which additionally replicated in UK Biobank, increasing confidence in our results. Larger sample sizes are necessary to identify novel genes using a strict multiple-testing threshold. Secondly, we do not have access to a dataset for comparing real and predicted gene expression in a HL population (as such a publicly available dataset does not, to our knowledge, currently exist); also, the tissue used for GENOA gene expression quantification, LCLs, differs from that in MESA (monocytes) and DGN (whole blood). Comparisons in GENOA are thus complicated by tissue heterogeneity, though we note that prior studies have performed comparisons (for example across eQTLs) across these relatively similar blood cell related tissues (for example LCLs, whole blood, and peripheral blood mononuclear cells are compared in the eQTLGen meta-analysis [57]). Thirdly, gene with cross-validation *R*^2^ < 1% and passing lenient significant threshold (Table 1 and Supplementary Table S4) might be derived from potential GWAS SNPs that have relatively large eQTL effect sizes, rather than implicate causality in that they may not indicate genetic effect on phenotype mediated through gene expression. In addition, we note that cohorts were classified as HL and AA based primarily on participant self-report; these self-identified race/ethnicity groupings, which are socially defined, encompass a variety of genetic ancestry backgrounds. Therefore, exact ancestry matching between, for example, MESA HL participants and HL participants in cohorts included in the hematological traits meta-analysis cannot be assumed.

In summary, TWAS analysis can help to enhance our understanding of the genetics of complex traits, including hematological indices. TWAS not only helps to identify potential causal genes at known GWAS loci, but can also increase power for new candidate gene and locus discovery. Some gene-trait associations in our analysis were only revealed by ancestry-matched TWAS reference panels due to the difference in MAF of included variants for expression prediction across populations. More work is needed to both generate large expression datasets in non-European-ancestry populations and apply these datasets to TWAS discovery for complex traits across ancestrally diverse cohorts.

## Figures and Tables

**Table 1:** The table shows the marginally significant results discovered by MESA HL for all four phenotypes. “NA” denotes there is no known variants +/− 1 MB around the gene.

Gene	Chr	Start_hg38	End_hg38	Phenotype	Meta_beta	Meta_se	Direction	Marginal *p*-Value	Conditional *p*-Value	Model *R*^2^	Cross-Validation *R*^2^	TWAS Reference Panel	Discovery Population
ADAM15	1	155050566	155062775	HCT	−0.078	0.018	(−−−−+)	8.74 $\times 10^{-6}$	2.52 $\times 10^{-1}$	0.301	0.395	MESA	HL
THBS3	1	155195588	155209051	HCT	0.212	0.039	(+++++)	5.40 $\times 10^{-8}$	9.22 $\times 10^{-1}$	0.1	0.060	MESA	HL
GTF2IRD2B	7	75092573	75149817	HCT	−0.249	0.056	(−−−−−)	9.18 $\times 10^{-6}$	NA	0.138	0.004	MESA	HL
AGAP6	10	49982190	50010499	HCT	−0.231	0.059	(−−−−−)	8.44 $\times 10^{-5}$	NA	0.119	0.017	MESA	HL
SMAD6	15	66702228	66782849	HCT	−0.356	0.081	(−−−−+)	1.14 $\times 10^{-5}$	8.55 $\times 10^{-6}$	0.074	0.005	MESA	HL
ADAM15	1	155050566	155062775	HGB	−0.066	0.018	(−−−−+)	3.95 $\times 10^{-5}$	1.51 $\times 10^{-1}$	0.301	0.395	MESA	HL
THBS3	1	155195588	155209051	HGB	0.159	0.039	(++++-)	2.88 $\times 10^{-5}$	7.71 $\times 10^{-1}$	0.1	0.060	MESA	HL
ARHGAP19	10	97222173	97292673	HGB	−0.215	0.056	(−−−−+)	7.15 $\times 10^{-5}$	NA	0.1	0.011	MESA	HL
CCDC15	11	124954121	125041489	HGB	0.059	0.016	(++++−)	5.84 $\times 10^{-5}$	NA	0.342	0.235	MESA	HL
SMAD6	15	66702228	6678284	HGB	−0.344	0.080	(−−−−+)	9.0 $\times 10^{-6}$	4.77 $\times 10^{-5}$	0.074	0.005	MESA	HL
IL6R	1	154405193	154469450	PLT	−0.232	0.058	(−−−−+)	6.06 $\times 10^{-5}$	7.63 $\times 10^{-1}$	0.065	0.014	MESA	HL
BAK1	6	33572547	33580293	PLT	−0.118	0.029	(−−−+−)	4.95 $\times 10^{-5}$	2.42 $\times 10^{-1}$	0.167	0.088	MESA	HL
PAQR8	6	52361421	52407777	PLT	0.080	0.019	(+++++)	4.79 $\times 10^{-5}$	1.27 $\times 10^{-3}$	0.268	0.165	MESA	HL
TNFAIP2	14	103123442	103137439	PLT	−0.265	0.065	(−−−−−)	6.85 $\times 10^{-5}$	8.10 $\times 10^{-3}$	0.095	0.013	MESA	HL
SLC22A4	5	132294394	132344190	WBC	0.117	0.027	(+++++)	1.73 $\times 10^{-5}$	1.53 $\times 10^{-1}$	0.172	0.126	MESA	HL
BAK1	6	33572547	33580293	WBC	−0.110	0.028	(−−−−+)	9.47 $\times 10^{-5}$	1.46 $\times 10^{-4}$	0.167	0.088	MESA	HL
GRINA	8	143990056	143993415	WBC	−0.298	0.077	(−−+−−)	9.70 $\times 10^{-5}$	NA	0.066	0.004	MESA	HL
ATXN2	12	111443485	111599676	WBC	−0.338	0.071	(−−+−+)	1.56 $\times 10^{-6}$	2.12 $\times 10^{-3}$	0.079	0.003	MESA	HL

## Data Availability

Data used in these analyses can be obtained either through the coordinating centers of the participating cohort and biobank studies or through dbGaP. Relevant dbGaP accession numbers are listed below. BioMe phs000227 (MEGA) and phs000925; ARIC phs000557; CARDIA phs000613; WHI phs000227 (MEGA) and phs000386; HCHS/SOL phs000810; GERA phs000674; MESA phs001416, phs000209; GENOA phs000379.

## Supplementary Materials

The following are available online at https://www.mdpi.com/article/10.3390/genes12071049/s1, Supplementary Figure S1: Detailed Study Overview; Supplementary Figure S2: Ancestry Inference Results. Global ancestry estimates for each cohort were summarized in violin plots; Supplementary Figure S3: Comparison of well-predicted genes between and MESA AA reference panels, at different true R2 thresholds; Supplementary Figure S4: Mirror plots for TWAS and GWAS results; Supplementary Figure S5: Model *R*^2^ vs. cross-validation *R*^2^. Supplementary Table S1: Summary of eQTL and GWAS datasets used for this study; Supplementary Table S2: Comparison of training results between DGN and MESA reference panel; Supplementary Table S3: Comparison of performance of prediction of true gene expression in GENOA using DGN and MESA AA reference panels; Supplementary Table S4: All the association results identified by marginal *p*-value < 1 × 10^−4^. Supplementary Table S5: All the known GWAS variants used for conditional analysis; Supplementary Table S6: The 8 multi-gene loci results from our TWAS analyses, with fine-mapping credible set posterior probabilities of inclusion; Supplementary Table S7: Bonferroni corrected *p*-value thresholds for TWAS analysis in each ancestry group and with each reference panel, based on the number of tested genes.
