# Supplementary material for: Transcriptome-Wide Association Study of Blood Cell Traits in African Ancestry and Hispanic/Latino Populations
Source: Genes (Basel). 2021 Jul 8;12(7):1049. doi: 10.3390/genes12071049 (PMC8307403; doi:10.3390/genes12071049)

Supplementary Content

Included cohorts

Hispanic Community Health Study/Study of Latinos (HCHS/SOL) HCHS/SOL is a prospective, community-based cohort study of 16,415 self-identified Hispanic/Latino adults in Bronx, NY, Chicago, IL, Miami, FL, San Diego, CA, designed to identify risk factors for chronic diseases including CVD and diabetes, as well as pulmonary and sleep disorders. Recruitment was conducted based on a two-stage probability sample of households near each recruitment center, first defined by census block group and then sampled by household. Households with H/L surnames and adults over age 45 were oversampled to increase target population representation and achieve a balanced age distribution. Seventy-eight percent of participants (N=12,803) consented to provide DNA for research purposes and have been genotyped both on the Illumina Omni2.5M array (plus 150,000 custom SNPs, including ancestry-informative markers, Native American population specific variants, previously identified GWAS hits, and other candidate polymorphisms for a total of 2,293,715 SNPs) [1] and the Illumina Multi-Ethnic Genotyping Array (MEGA) array (containing a total of 1,705,969 SNPs) in efforts from the Population Architecture for Genetic Epidemiology (17) consortium to better assess variation in non-European populations. The MEGA array also includes additional exonic, functional, and clinically-relevant variants. Illumina 2.5M array genotypes were available for 12,803 samples, among whom 11,887 samples also had MEGA array genotypes. Imputation was performed on 11,588 samples with hematological trait data after merging Omni2.5M array genotypes and MEGA array genotypes.

Women's Health Initiative (WHI) WHI is a prospective, long-term, multicenter cohort study designed to investigate the postmenopausal health of women in the US, including risk for heart disease, breast and colon cancer, and osteoporosis. WHI originally enrolled 161,808 women aged 50-79 between 1993 and 1998 at 40 centers across the US, including both a clinical trial (including three trials for hormone therapy, dietary modification, and calcium/vitamin D) and an observational study arm [2]. WHI recruited a socio-demographically diverse population, with racial/ethnic minority group representation similar to all US women in this age range (~17% minority participation). Two WHI extension studies conducted additional follow-up on consenting women from 2005-2010 and 2010-2015. Genotyping was available on some WHI

participants through the WHI SNP Health Association Resource (SHLRe) resource, which used the Affymetrix 6.0 array (~906,600 SNPs, 946,000 copy number variation probes) and on other participants through the MEGA array [3]. Imputation and association analysis was performed separately in individuals with Affymetrix only, MEGA only, and both Affymetrix and MEGA data. For variants with both Affymetrix and MEGA genotypes available, MEGA genotypes were used. In total, 4,318 Hispanic/Latino and 8,494 AA women with blood cell traits were included.

UK Biobank The UK Biobank [4] recruited 500,000 people aged between 40-69 years in 2006-2010, establishing a prospective biobank study to understand risk factors for common diseases such as cancer, heart disease, stroke, diabetes, and dementia). Participants are being followed-up through health records from the UK National Health Service. UK Biobank has genotype data on all enrolled participants, as well as extensive baseline questionnaire and physical measures and stored blood and urine samples. Hematological traits were assayed as previously described [5]. Genotyping on custom Axiom arrays and subsequent quality control, as well as imputation for European ancestry participants, has been previously described [6].

Due to the large number of European participants in UK Biobank, samples were included in our replication analysis if identified as European through a combination of ancestry-self report and k-means clustering of genetic principal components in order to minimize genomic inflation due to population stratification. First, we calculated principal components and their loadings for all 488,377 genotyped UKBB participants using high quality variants in the UK Biobank data set that overlapped with the participants in the 1000G Phase 3 v5 (1KG) reference panel. Reference ancestries used included 504 European (EUR), 347 American Admixed (AMR) 661 African (AFR), 504 East Asian (EAS) and 489 South Asian (SAS) samples (overall 2,504). We projected the 1KG reference panel dataset on the calculated PCA loadings from UKBB. We then used k-means clustering with 4 dimensions, defined by the first 4 PCs, to identify the individuals that clustered with the majority of 1KG reference panels in each ancestry. We used self-reported ancestry/ethnicity (variable "ethnic_background"), in some circumstances, to adjust these groups. UKBB participants defined as European ancestry include those that cluster with the most 1KG Europeans (EA) by k-means clustering. We adjusted this group by removing those that self-reported as Indian, Pakistani, Bangladeshi, Any other Asian background, Black or Black British, Caribbean, African, Any other Black background, or Chinese (N=32), due to the

possibility of a sample swap. Additionally, we removed any individuals with self-reported mixed ancestry from the European focused analyses. A total of 451,305 remained in the European ancestry group. For the African ancestry subset used in our analysis, we included all individuals that cluster with the 1KG AFR samples by k-means clustering, except n=7 individuals self-reported as follows in variable Ethnic background (variable 21000-0.0), at baseline visit (due to the possibility of a sample swap): White, British, Irish, Any other White background, Indian, Pakistani, Bangladeshi, Any other Asian background, or Chinese. We also added to our African ancestry cluster those that did not cluster in a group using k-means, but self-reported White and Black Caribbean, White and Black African, Black or Black British, Caribbean, African, or Any other Black background (n=660). A total of 9354 participants remained in the African ancestry group. We used the UK Biobank provided imputation to the HRC, UK10K, and 1000 Genomes phase 3 reference panels for European ancestry subjects (Bycroft, 2018), and performed our own imputation to TOPMed freeze 5 for African ancestry subjects.

UK Biobank European samples were excluded from the replication analysis based on positive pregnancy status, drug treatments, cancer self-report, ICD9 and ICD10 disease codes for hematological related disorders, surgical procedures, or withdrawn consent. Samples were included in the replication cohort only if they had complete data for all covariates and phenotypes. In total, 399,835 samples were included in the European focused replication analysis with data for hematological indices, and 8,262 were included in the African ancestry focused discovery TWAS meta-analysis.

Genetic Epidemiology Research on Aging (GERA) The GERA cohort includes over 100,000 adults who are members of the Kaiser Permanente Medical Care Plan, Northern California Region (KPNC) and consented to research on the genetic and environmental factors that affect health and disease, linking together clinical data from electronic health records, survey data on demographic and behavioral factors, and environmental data with genetic data. The GERA cohort was formed by including all self-reported racial and ethnic minority participants with saliva samples (19%); the remaining participants were drawn sequentially and randomly from non-Hispanic White participants (81%). Genotyping was completed as previously described [7] using 4 different custom Affymetrix Axiom arrays with ethnic-specific content to increase

genomic coverage. Principal components analysis was used to characterize genetic structure in this multi-ethnic sample [8]. Blood cell traits were extracted from medical records. In individuals with multiple measurements, the first visit with complete white blood cell differential (if any) was used for each participant. Otherwise, the first visit was used. In total, 5,783 Hispanic/Latino and 2,246 AA participants with blood cell traits were included in the analysis.

Coronary Artery Risk Development in Young Adults (CARDIA) CARDIA is a longitudinal study of cardiovascular disease risk initiated in 1985-86 in 5,115 AA and European ancestry men and women aged 18-30 years. The CARDIA sample was recruited at four sites: Birmingham, AL, Chicago, IL, Minneapolis, MN, and Oakland, CA [9,10]. Genotyping was performed through the CARe consortium [11,12] using an Affymetrix 6.0 array. In total, 1,619 AA participants with blood cell traits were included in the analysis.

Atherosclerosis Risk in Communities (ARIC) The ARIC study was initiated in 1987 and recruited participants age 45-64 years from 4 field centers (Forsyth County, NC; Jackson, MS; northwestern suburbs of Minneapolis, MN; Washington County, MD) in order to study cardiovascular disease and its risk factors [13], including the participants of self-reported AA ancestry included here. Standardized physical examinations and interviewer-administered questionnaires were conducted at baseline (1987-89), three triennial follow-up examinations, a fifth examination in 2011-13, and a sixth exam in 2016-2017. Genotyping was performed through the CARe consortium Affymetrix 6.0 array [11,12]. In total, 2,392 AA participants with blood cell traits were included in the analysis.

BioMe Biobank The Charles Bronfman Institute for Personalized Medicine at the Mount Sinai Medical Center (MSMC), BioMeTM Biobank (BioMe) is an electronic medical record linked biospecimen repository of consented MSMC patient samples from and annual population of >70,000 inpatients and >800,000 outpatients. MSMC serves the diverse communities of Central Harlem (86% AA), East Harlem (88% H/L), and the Upper East Side (88% EA) of Manhattan. Between September 2007-August 2013, BioMeTM enrolled 26,500 participants (including 25% AA and 36% primarily Carribean H/L), using direct recruitment from over 30 clinical care site waiting areas.

Global Ancestry Inference

We ran RFMix [14] to infer the global ancestry for each study sample. For computational reasons, we used markers on chromosome 8 and chromosome 18, with RFMix's default parameter settings. We used a reference built from African, European samples in the 1000 Genomes Project and Native American samples from the Human Genome Diversity Project (HGDP). 92 samples were selected for each of the three ancestral populations, namely African, European, and Native American.

Reference

1. Conomos, M.P.; Laurie, C.A.; Stilp, A.M.; Gogarten, S.M.; McHugh, C.P.; Nelson, S.C.; Sofer, T.; Fernandez-Rhodes, L.; Justice, A.E.; Graff, M.; et al. Genetic Diversity and Association Studies in US Hispanic/Latino Populations: Applications in the Hispanic Community Health Study/Study of Latinos. *Am J Hum Genet* **2016**, *98*, 165-184, doi:10.1016/j.ajhg.2015.12.001.
2. The Women's Health Initiative Study Group. Design of the Women's Health Initiative clinical trial and observational study. *Controlled clinical trials* **1998**, *19*, 61-109.
3. Wojcik, G.L.; Graff, M.; Nishimura, K.K.; Tao, R.; Haessler, J.; Gignoux, C.R.; Highland, H.M.; Patel, Y.M.; Sorokin, E.P.; Avery, C.L.; et al. Genetic analyses of diverse populations improves discovery for complex traits. *Nature* **2019**, *570*, 514-518, doi:10.1038/s41586-019-1310-4.
4. UK Biobank. UK Biobank: rationale, design and development of a large-scale prospective resource. Available online: http://www.ukbiobank.ac.uk/resources/ (accessed on
5. Astle, W.J.; Elding, H.; Jiang, T.; Allen, D.; Ruklisa, D.; Mann, A.L.; Mead, D.; Bouman, H.; Riveros-Mckay, F.; Kostadima, M.A.; et al. The Allelic Landscape of Human Blood Cell Trait Variation and Links to Common Complex Disease. *Cell* **2016**, *167*, 1415-1429 e1419, doi:10.1016/j.cell.2016.10.042.
6. Bycroft, C.; Freeman, C.; Petkova, D.; Band, G.; Elliott, L.T.; Sharp, K.; Motyer, A.; Vukcevic, D.; Delaneau, O.; O'Connell, J.; et al. The UK Biobank resource with deep phenotyping and genomic data. *Nature* **2018**, *562*, 203-209, doi:10.1038/s41586-018-0579-z.
7. Kvale, M.N.; Hesselson, S.; Hoffmann, T.J.; Cao, Y.; Chan, D.; Connell, S.; Croen, L.A.; Dispensa, B.P.; Eshragh, J.; Finn, A.; et al. Genotyping Informatics and Quality Control for 100,000 Subjects in the Genetic Epidemiology Research on Adult Health and Aging (GERA) Cohort. *Genetics* **2015**, *200*, 1051-1060, doi:10.1534/genetics.115.178905.
8. Banda, Y.; Kvale, M.N.; Hoffmann, T.J.; Hesselson, S.E.; Ranatunga, D.; Tang, H.; Sabatti, C.; Croen, L.A.; Dispensa, B.P.; Henderson, M.; et al. Characterizing Race/Ethnicity and Genetic Ancestry for 100,000 Subjects in the Genetic Epidemiology Research on Adult Health and Aging (GERA) Cohort. *Genetics* **2015**, *200*, 1285-1295, doi:10.1534/genetics.115.178616.
9. Friedman, G.D.; Cutter, G.R.; Donahue, R.P.; Hughes, G.H.; Hulley, S.B.; Jacobs, D.R., Jr.; Liu, K.; Savage, P.J. CARDIA: study design, recruitment, and some characteristics of the examined subjects. *J Clin Epidemiol* **1988**, *41*, 1105-1116.

10. Cutter, G.R.; Burke, G.L.; Dyer, A.R.; Friedman, G.D.; Hilner, J.E.; Hughes, G.H.; Hulley, S.B.; Jacobs, D.R., Jr.; Liu, K.; Manolio, T.A.; et al. Cardiovascular risk factors in young adults. The CARDIA baseline monograph. *Controlled clinical trials* **1991**, *12*, 1S-77S.
11. Musunuru, K.; Lettre, G.; Young, T.; Farlow, D.N.; Pirruccello, J.P.; Ejebe, K.G.; Keating, B.J.; Yang, Q.; Chen, M.H.; Lapchyk, N.; et al. Candidate gene association resource (CARe): design, methods, and proof of concept. *Circulation. Cardiovascular genetics* **2010**, *3*, 267-275, doi:10.1161/circgenetics.109.882696.
12. Lettre, G.; Palmer, C.D.; Young, T.; Ejebe, K.G.; Allayee, H.; Benjamin, E.J.; Bennett, F.; Bowden, D.W.; Chakravarti, A.; Dreisbach, A.; et al. Genome-wide association study of coronary heart disease and its risk factors in 8,090 African Americans: the NHLBI CARe Project. *PLoS genetics* **2011**, *7*, e1001300, doi:10.1371/journal.pgen.1001300.
13. The Atherosclerosis Risk in Communities (ARIC) Study: design and objectives. The ARIC investigators. *American journal of epidemiology* **1989**, *129*, 687-702.
14. Maples, Brian K.; Gravel, S.; Kenny, Eimear E.; Bustamante, Carlos D. RFMix: A Discriminative Modeling Approach for Rapid and Robust Local-Ancestry Inference. The American Journal of Human Genetics **2013**, 93, 278-288, doi:http://dx.doi.org/10.1016/j.ajhg.2013.06.020.

Supplementary Figures

Supplementary Figure S1. 1 Detailed Study Overview.

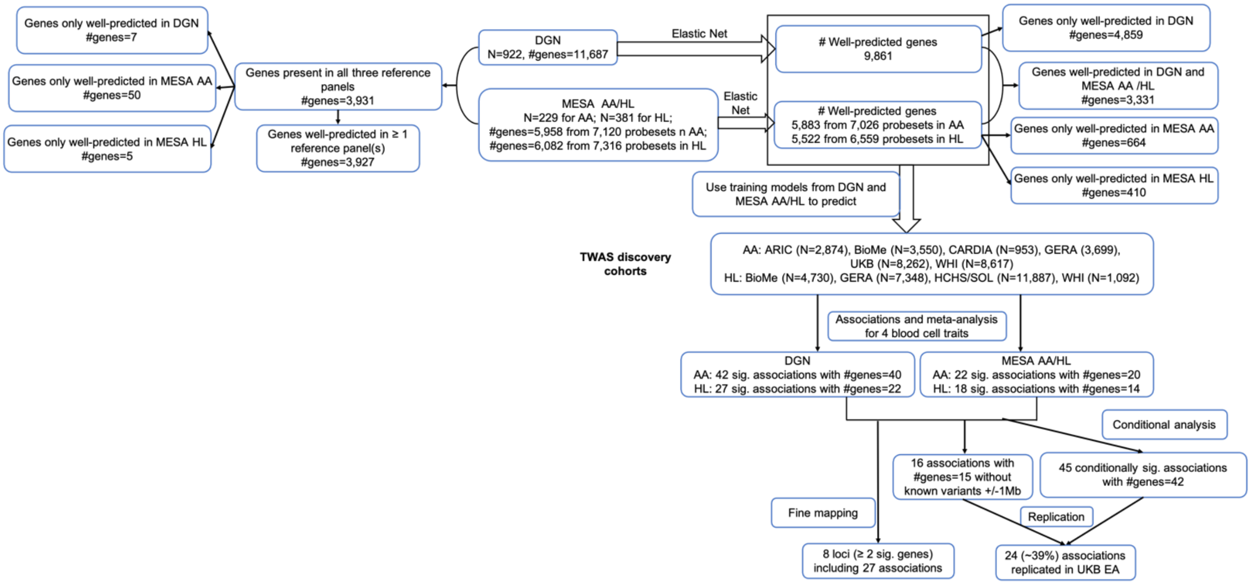

Supplementary Figure S2. Ancestry Inference Results. Global ancestry estimates for each cohort were summarized in violin plots. The red dots denote the median and the blue triangles denote the mean.

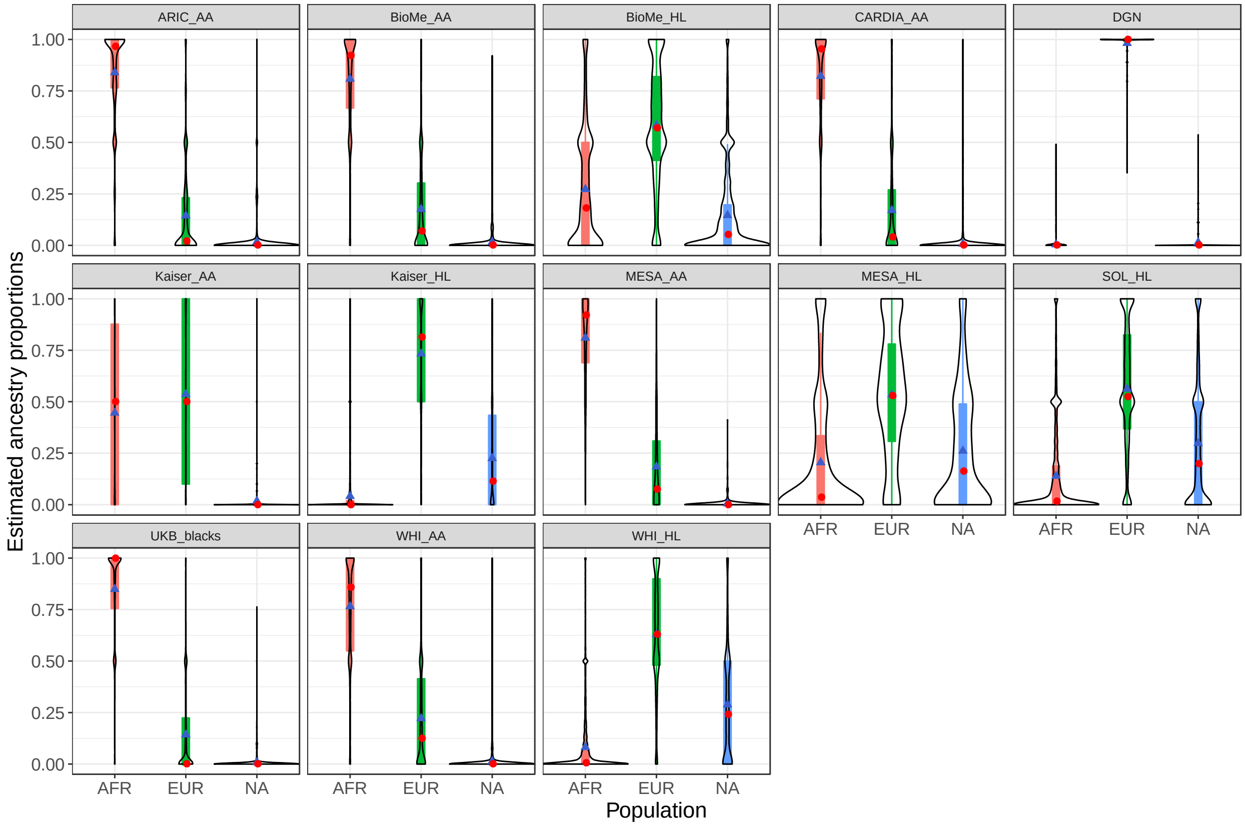

Supplementary Figure S3. Comparison of well-predicted genes between and MESA AA reference panels, at different true R2 thresholds. a-d. Venn diagrams of well-predicted genes (at four different thresholds: true R2 > 0.10, 0.15, 0.20 and 0.25 respectively) between DGN and MESA AA reference panels, when assessed in GENOA. e. Line plot shows how the proportions of shared or specific genes change with true R2 threshold values.

a. true R^2 > 0.10

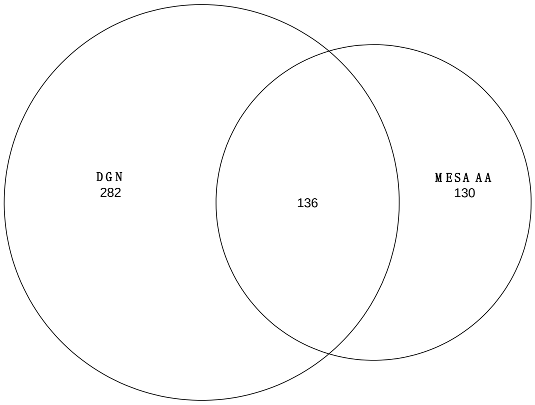

b. true R^2 > 0.15

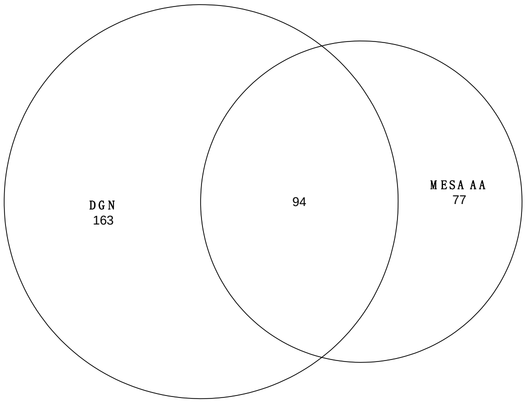

c. true R^2 > 0.20

d. true R^2 > 0.25

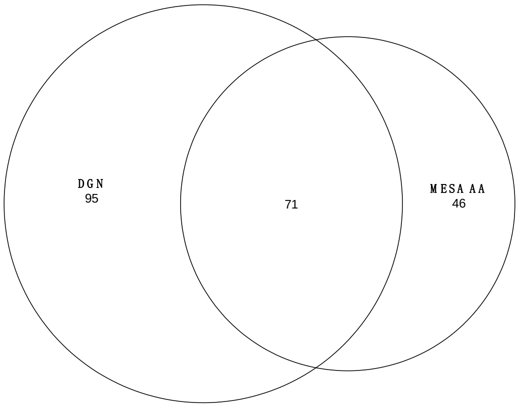

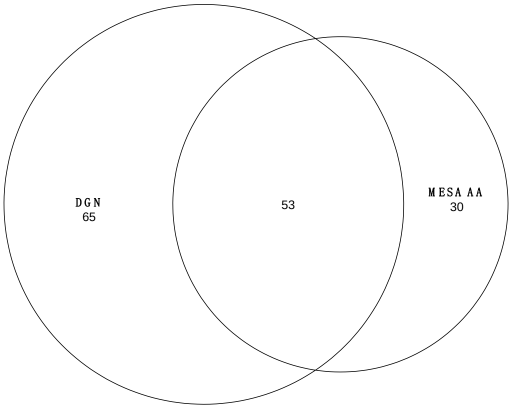

e

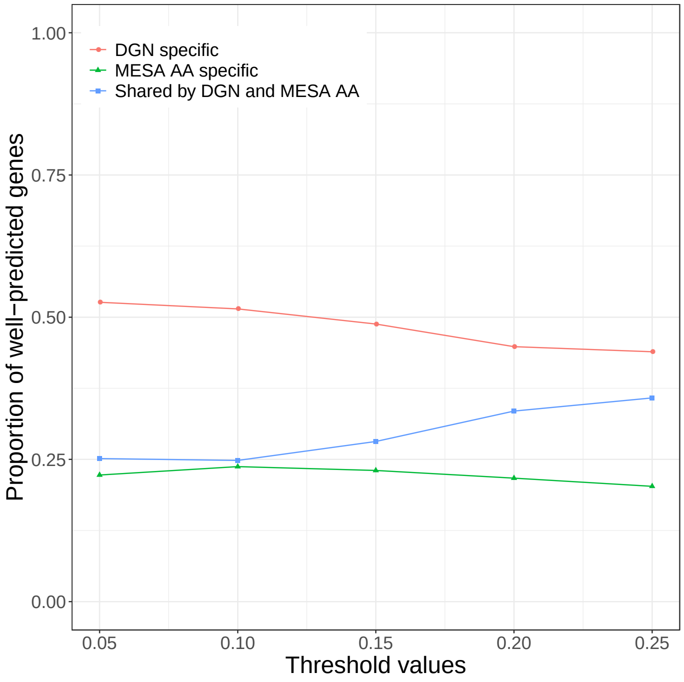

Supplementary Figure S4. Mirror plots for TWAS and GWAS results. The upper panel shows TWAS marginal results and the bottom panel shows GWAS results, both from meta-analyses, for African ancestry (AA) and Hispanic/Latino (HL) cohorts. The red dotted line denotes the significant threshold value: 1×10^{-4} for TWAS and 5×10^{-8} for GWAS.

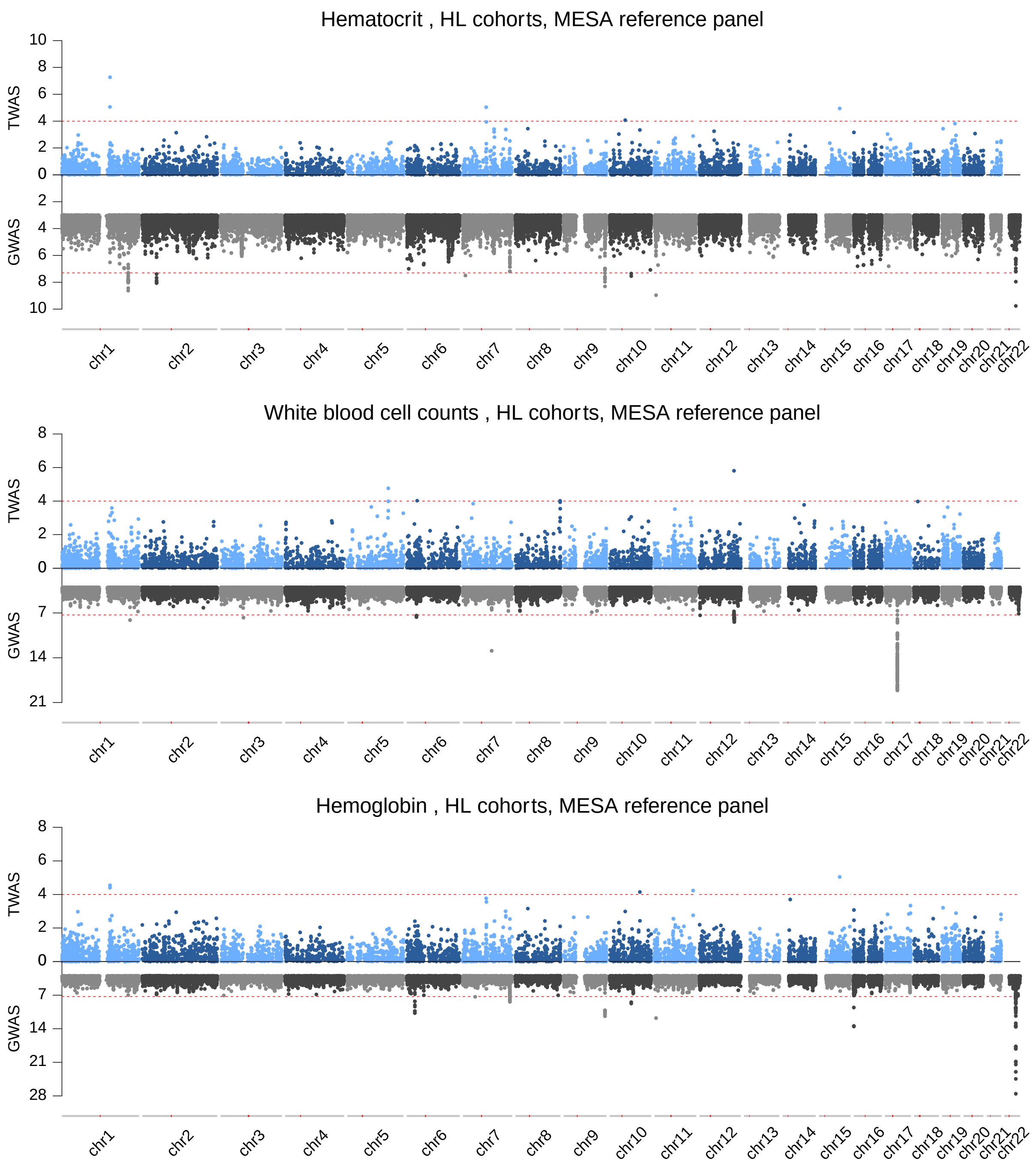

Hematocrit , HL cohorts, MESA reference panel
TWAS
GWAS
10
8
6
4
2
0
2
4
6
8
10
chr1
chr2
chr3
chr4
chr5
chr6
chr7
chr8
chr9
chr10
chr11
chr12
chr13
chr14
chr15
chr16
chr17
chr18
chr19
chr20
chr21
chr22
White blood cell counts , HL cohorts, MESA reference panel
TWAS
GWAS
8
6
4
2
0
7
14
21
Hemoglobin , HL cohorts, MESA reference panel
TWAS
GWAS
8
6
4
2
0
7
14
21
28

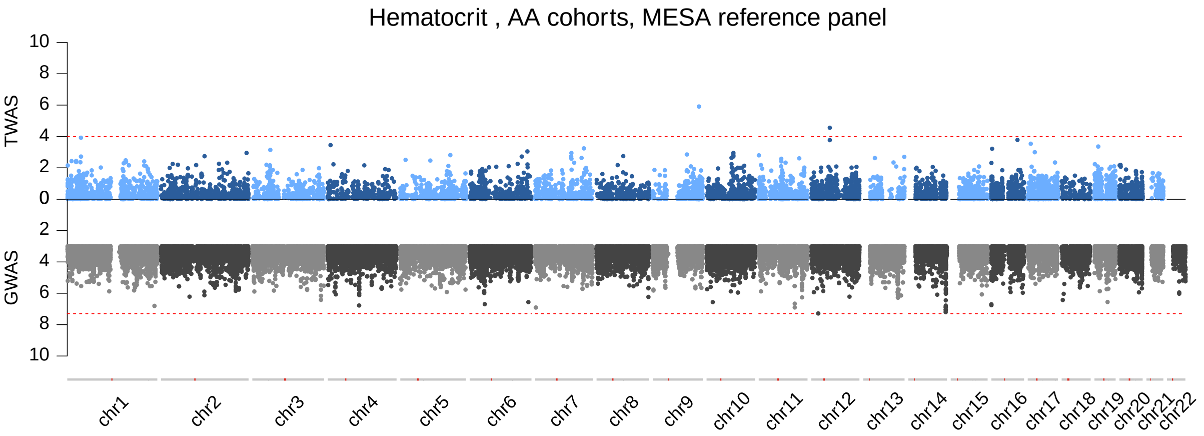
Hematocrit , AA cohorts, MESA reference panel
TWAS
GWAS
10
8
6
4
2
0
2
4
6
8
10
chr1
chr2
chr3
chr4
chr5
chr6
chr7
chr8
chr9
chr10
chr11
chr12
chr13
chr14
chr15
chr16
chr17
chr18
chr19
chr20
chr21
chr22

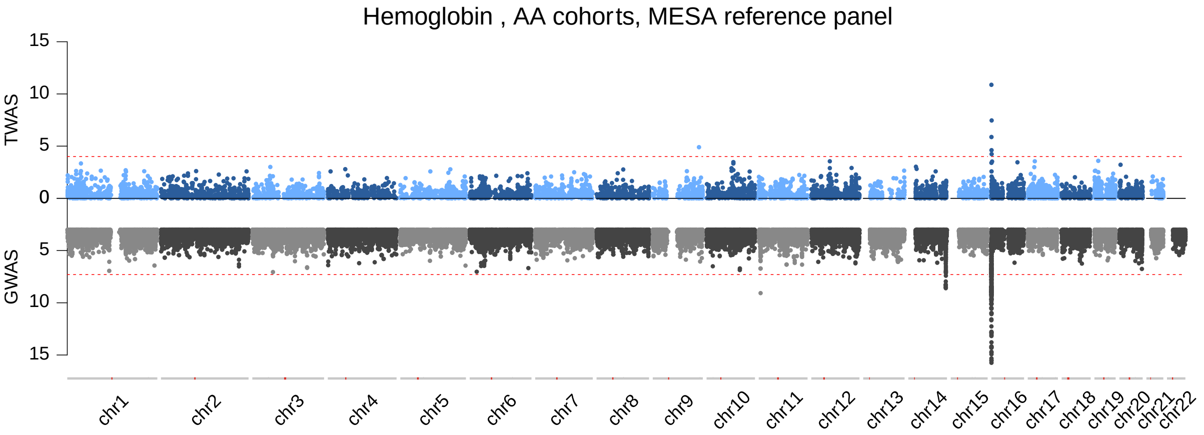
Hemoglobin , AA cohorts, MESA reference panel
TWAS
GWAS
15
10
5
0
5
10
15
chr1
chr2
chr3
chr4
chr5
chr6
chr7
chr8
chr9
chr10
chr11
chr12
chr13
chr14
chr15
chr16
chr17
chr18
chr19
chr20
chr21
chr22

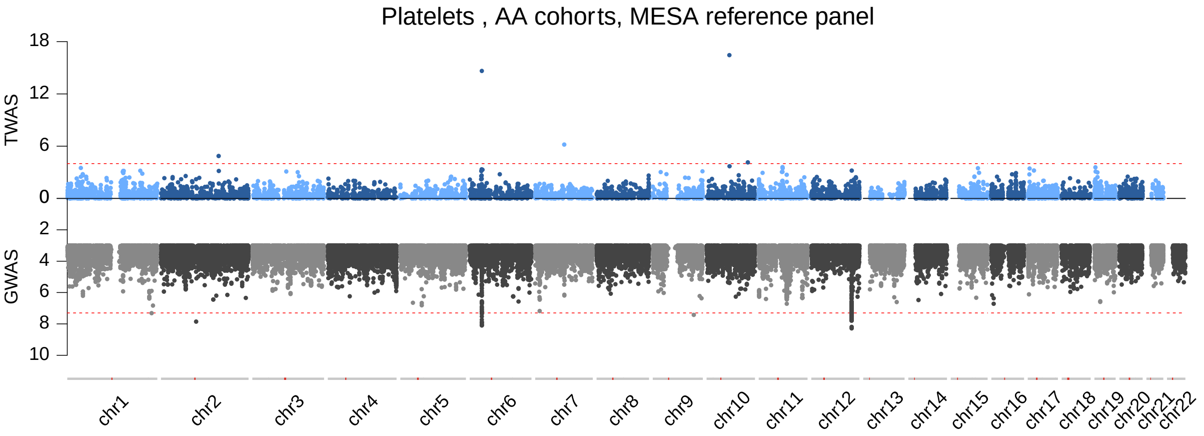
Platelets , AA cohorts, MESA reference panel
TWAS
GWAS
18
12
6
0
2
4
6
8
10
chr1
chr2
chr3
chr4
chr5
chr6
chr7
chr8
chr9
chr10
chr11
chr12
chr13
chr14
chr15
chr16
chr17
chr18
chr19
chr20
chr21
chr22

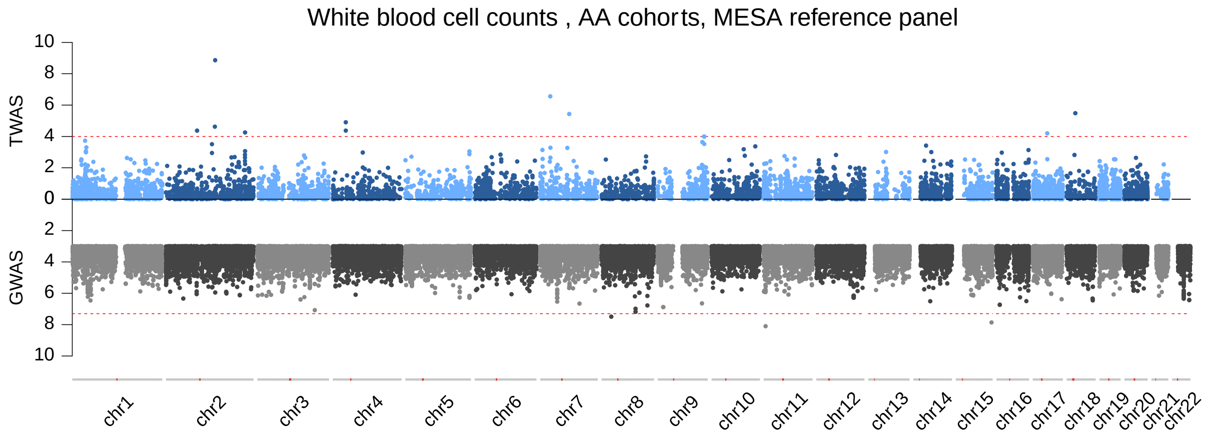
White blood cell counts , AA cohorts, MESA reference panel
TWAS
GWAS
10
8
6
4
2
0
2
4
6
8
10
chr1
chr2
chr3
chr4
chr5
chr6
chr7
chr8
chr9
chr10
chr11
chr12
chr13
chr14
chr15
chr16
chr17
chr18
chr19
chr20
chr21
chr22

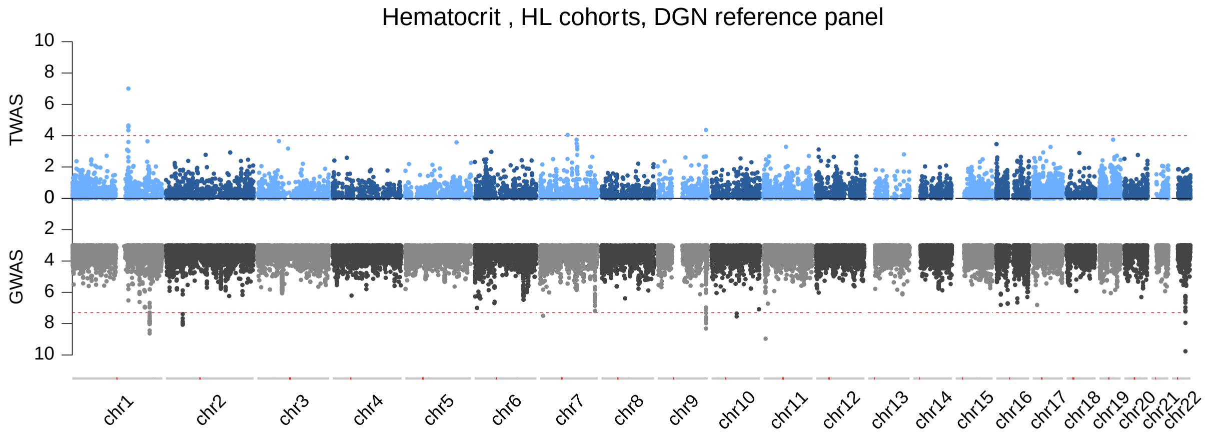
Hematocrit , HL cohorts, DGN reference panel
TWAS
GWAS
10
8
6
4
2
0
2
4
6
8
10
chr1
chr2
chr3
chr4
chr5
chr6
chr7
chr8
chr9
chr10
chr11
chr12
chr13
chr14
chr15
chr16
chr17
chr18
chr19
chr20
chr21
chr22

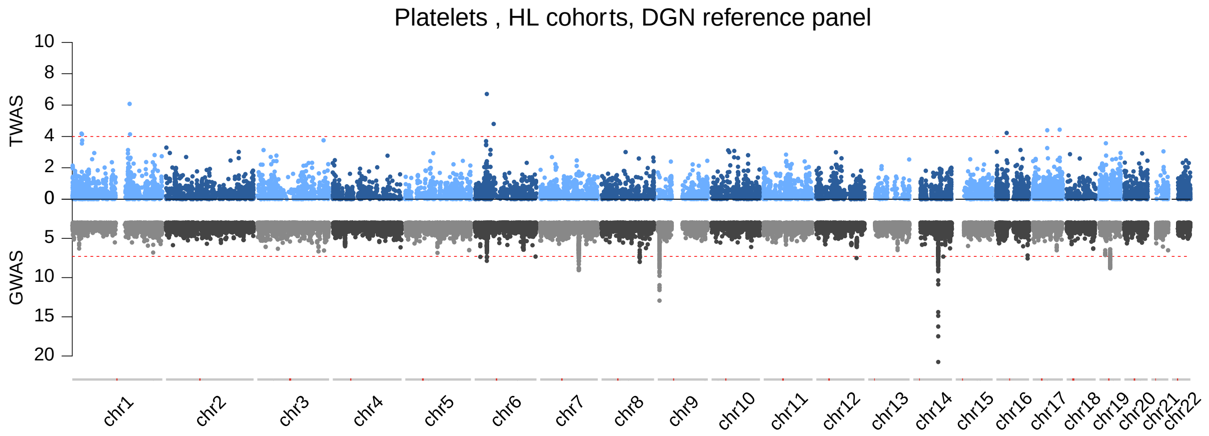
Platelets , HL cohorts, DGN reference panel
TWAS
GWAS
10
8
6
4
2
0
5
10
15
20
chr1
chr2
chr3
chr4
chr5
chr6
chr7
chr8
chr9
chr10
chr11
chr12
chr13
chr14
chr15
chr16
chr17
chr18
chr19
chr20
chr21
chr22

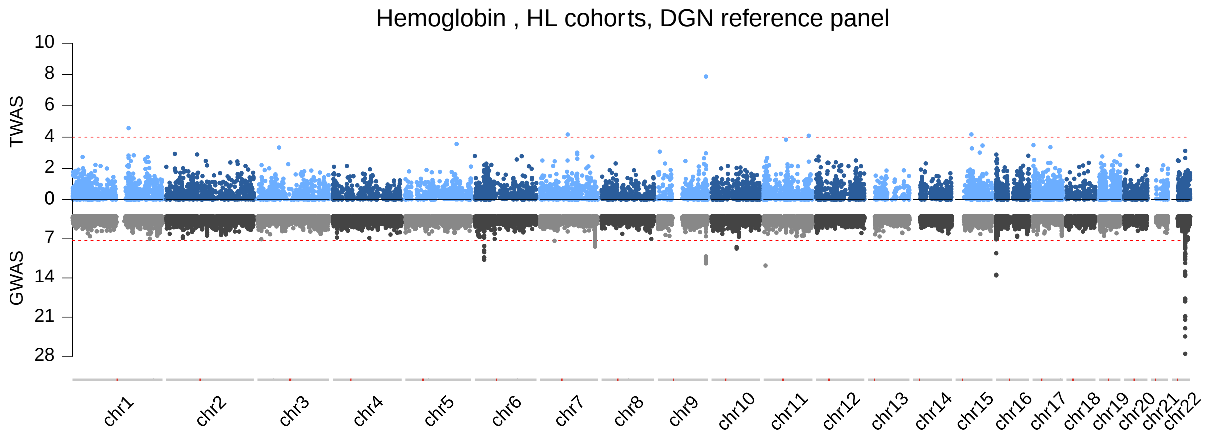
Hemoglobin , HL cohorts, DGN reference panel
TWAS
GWAS
10
8
6
4
2
0
7
14
21
28
chr1
chr2
chr3
chr4
chr5
chr6
chr7
chr8
chr9
chr10
chr11
chr12
chr13
chr14
chr15
chr16
chr17
chr18
chr19
chr20
chr21
chr22

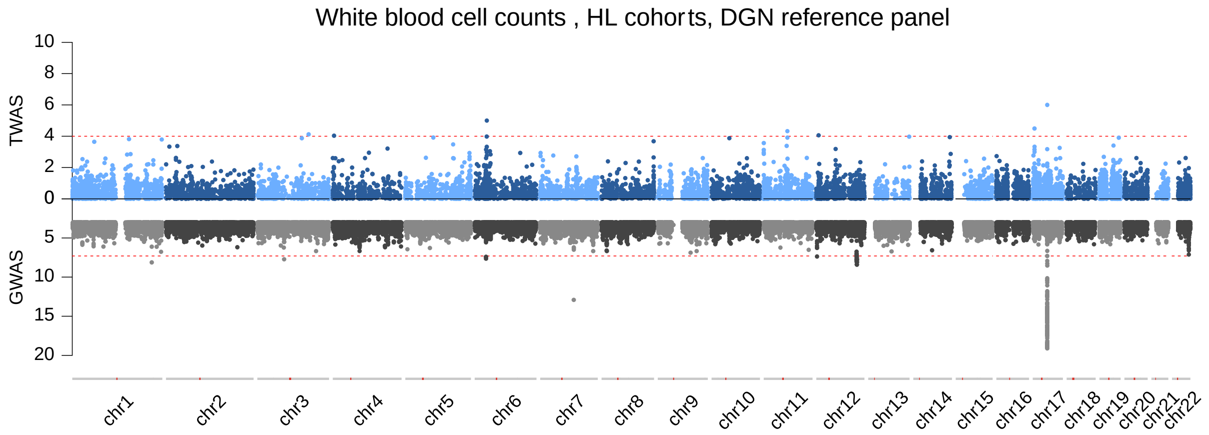
White blood cell counts , HL cohorts, DGN reference panel
TWAS
GWAS
10
8
6
4
2
0
5
10
15
20
chr1
chr2
chr3
chr4
chr5
chr6
chr7
chr8
chr9
chr10
chr11
chr12
chr13
chr14
chr15
chr16
chr17
chr18
chr19
chr20
chr21
chr22

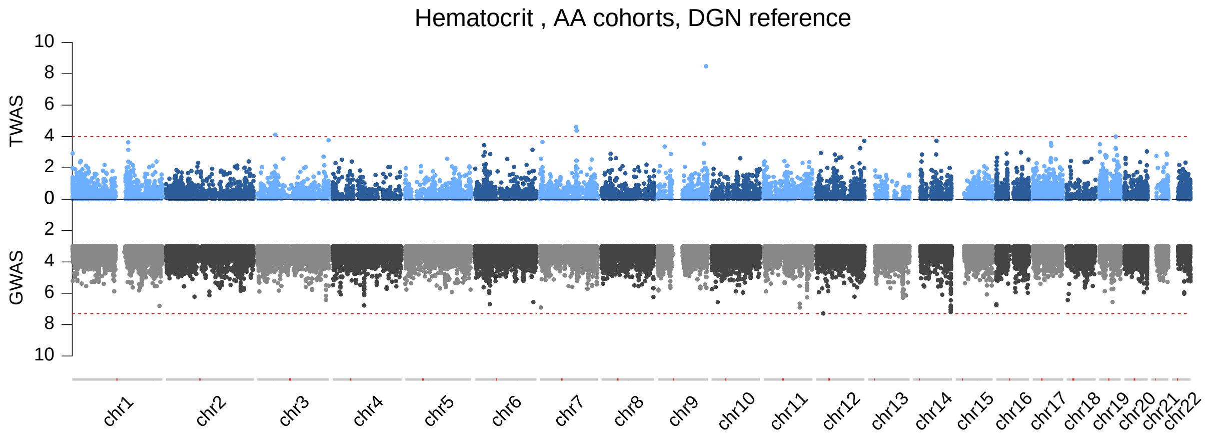

Hematocrit , AA cohorts, DGN reference
TWAS
GWAS
10
8
6
4
2
0
2
4
6
8
10
chr1
chr2
chr3
chr4
chr5
chr6
chr7
chr8
chr9
chr10
chr11
chr12
chr13
chr14
chr15
chr16
chr17
chr18
chr19
chr20
chr21
chr22

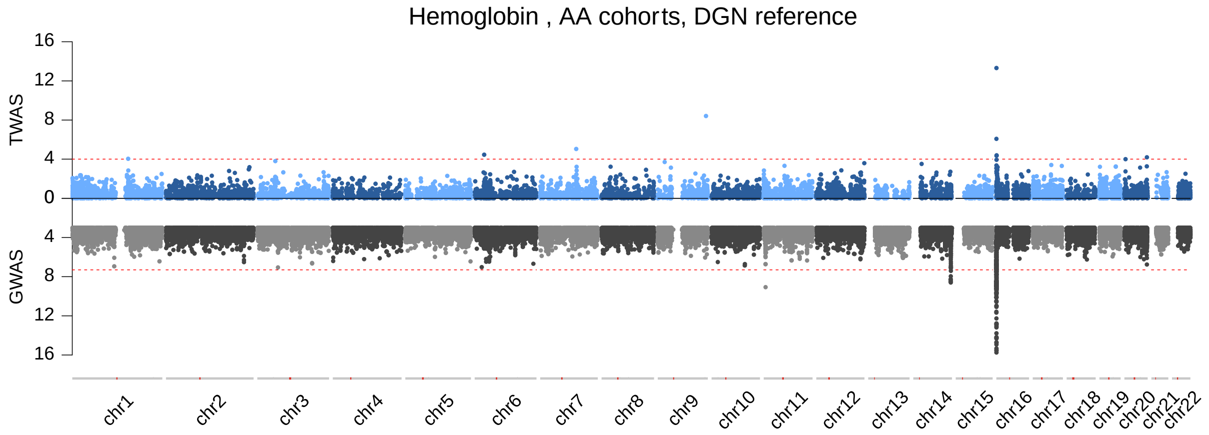

Hemoglobin , AA cohorts, DGN reference
TWAS
GWAS
16
12
8
4
0
4
8
12
16
chr1
chr2
chr3
chr4
chr5
chr6
chr7
chr8
chr9
chr10
chr11
chr12
chr13
chr14
chr15
chr16
chr17
chr18
chr19
chr20
chr21
chr22

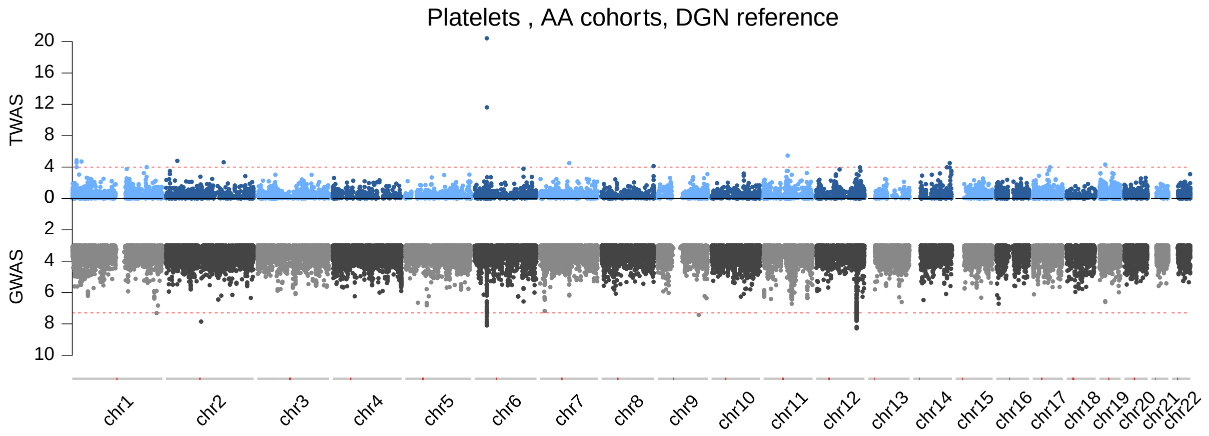

Platelets , AA cohorts, DGN reference
TWAS
GWAS
20
16
12
8
4
0
2
4
6
8
10
chr1
chr2
chr3
chr4
chr5
chr6
chr7
chr8
chr9
chr10
chr11
chr12
chr13
chr14
chr15
chr16
chr17
chr18
chr19
chr20
chr21
chr22

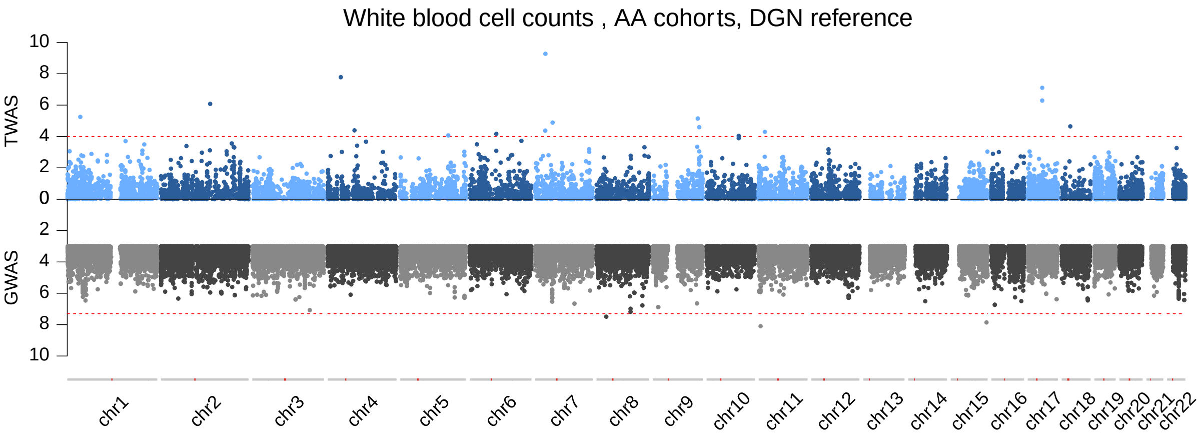

Supplementary Figure S5. **Model R^2 vs. cross-validation R^2**. The blue line denotes the fitting line, and the red line denotes the diagonal line. R is the Pearson correlation coefficient. The dots on the figure denote marginally significant genes from TWAS analysis.

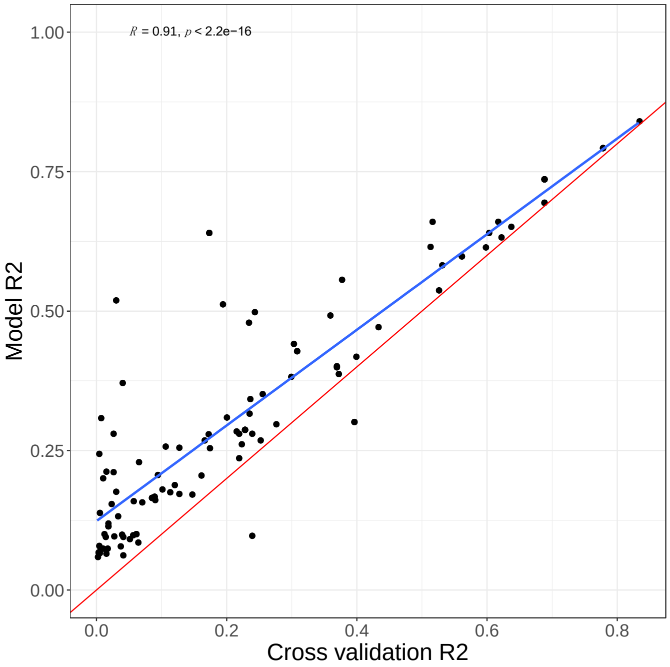

Supplement: Supplementary file 1 [file genes-12-01049-s001.zip › SupplementContent_Figures_finalSUB_R2.pdf]
